# The terpene synthase (TPS) gene family in kiwifruit shows high functional redundancy and a subset of TPS likely fulfil overlapping functions in fruit flavour, floral bouquet and defence

**DOI:** 10.1186/s43897-023-00057-0

**Published:** 2023-05-08

**Authors:** Wu Wang, Mindy Y. Wang, Yunliu Zeng, Xiuyin Chen, Xiaoyao Wang, Anne M. Barrington, Jianmin Tao, Ross G. Atkinson, Niels J. Nieuwenhuizen

**Affiliations:** 1 The New Zealand Institute for Plant and Food Research Ltd (PFR), Private Bag 92169, Auckland, New Zealand; 2 Institute of Botany, Jiangsu Province and Chinese Academy of Sciences, Nanjing, 210014 China; 3 Key Laboratory of Horticultural Plant Biology, College of Horticulture and Forestry Science, Huazhong Agricultural University, Wuhan, 430070 People’s Republic of China; 4 College of Horticulture, Nanjing Agricultural University, Nanjing, 210095 China

**Keywords:** *Actinidia*, Aroma, Genome analysis, Monoterpene, Sesquiterpene, Volatile

## Abstract

Volatile terpenes are important compounds that influence fruit flavour and aroma of kiwifruit. Terpenes in plants also impact on the floral bouquet and defence against pests and pathogens in leaves and fruit. To better understand the overlapping roles that terpenes may fulfil in plants, a systematic gene, chemical and biochemical analysis of terpenes and terpene synthases (TPS) was undertaken in Red5 kiwifruit (*Actinidia* spp.). Analysis of the Red5 genome shows it contains only 22 TPS gene models, of which fifteen encode full-length TPS. Thirteen TPS can account for the major terpene volatiles produced in different tissues of Red5 kiwifruit and in response to different stimuli. The small Red5 TPS family displays surprisingly high functional redundancy with five TPS producing linalool/nerolidol. Treatment of leaves and fruit with methyl jasmonate enhanced expression of a subset of defence-related TPS genes and stimulated the release of terpenes. Six TPS genes were induced upon herbivory of leaves by the economically important insect pest *Ctenopseustis obliquana* (brown-headed leaf roller) and emission, but not accumulation, of (*E*)- and (*Z*)-nerolidol was strongly linked to herbivory. Our results provide a framework to understand the overlapping biological and ecological roles of terpenes in *Actinidia* and other horticultural crops.

**Supplementary Information:**

The online version contains supplementary material available at 10.1186/s43897-023-00057-0.

## Core

Molecular, chemical and biochemical analysis of TPS genes in kiwifruit is used to decipher the volatile language of terpenes and their overlapping roles in fruit flavour, floral bouquet, and defence. Thirteen terpene synthases were identified in the genome of kiwifruit and can account for the major volatile terpenes in fruit, flowers as well as vegetative tissues. We provide a framework to understand the overlapping biological and ecological roles of terpenes in *Actinidia* and other horticultural crops.

## Gene & Accession Numbers (GenBank)

*AcNES2* (OM884050), *AcNES3* (OM884051), *AcBCS* (OM884052), *AcGES* (OM884053), *AcLIS/NES* (OM884054).

## Introduction

Terpene hydrocarbons and terpenoids (oxygenated terpenes) embody a wide and functionally diversified set of natural metabolites that play important roles in general and specialised physiological, biochemical and biological processes including plant growth and development (e.g. production of phytohormones and pigments), interaction with plant pollinators, involvement in direct and indirect defences, adding flavour and aroma to herbs and fruit, and by acting as anti-oxidants (Dudareva et al. 2013; Gershenzon and Dudareva 2007; Tholl 2015). Plant terpenes can be classified based on the number of isoprene units (C_5_H_8_) they contain into hemiterpenes e.g. isoprene (C5), monoterpenes (C10), sesquiterpenes (C15), diterpenes (C20), sesterterpenes (C25) and triterpenes (C30). The diversity of observed terpene compounds changes in response to environmental stimuli and their emission is highly regulated, both spatially and temporally (Dudareva et al. 2013). Biosynthesis of specialised terpenes in plants is often restricted to specific tissues, such as flowers (Bao et al. 2020; Gao et al. 2018), roots (Vaughan et al. 2013; Yahyaa et al. 2015) and leaves, stems as well as fruits (Courtois et al. 2012; Yu et al. 2020). Terpenes can also be stored in specialised organs such as trichomes and oil glands (Wang et al. 2008).

Kiwifruit (*Actinidia* spp.) is one of the most recently domesticated fruit crops, and favoured by consumers for its flavour and nutritional properties (Richardson et al. 2018). A number of abundant terpenoids have been identified in *Actinidia* flowers such as linalool, germacrene D, (*E*)-nerolidol and (*E*,*E*)-α-farnesene, and four TPS genes responsible for their production functionally characterised (Chen et al. 2010; Green et al. 2012; Nieuwenhuizen et al. 2009). Terpene compounds in the ripe fruit of *A. arguta* and a range of *A. chinensis* cultivars including ‘Hayward’, ‘Hort16A’ and ‘Hongyang’ have been reported (Du et al. 2019; Garcia et al. 2012; Nieuwenhuizen et al. 2015). A cluster of TPS genes on chromosome 29 that is responsible for the production of terpinolene in ripe *A. arguta* ‘Hortgem Tahi’ fruit (*AaTPS1*) and the signature flavour compound 1,8-cineole in ripe ‘Hort16A’ fruit (*AcTPS1b*) has been identified. Allelic variation at this locus is a target for breeding fruit with improved aroma and flavour (Nieuwenhuizen et al. 2015; Zeng et al. 2020).

The role of terpenoids in plant defence against pest and diseases has also received considerable attention in recent years. In response to insect herbivory, plants have evolved several defence mechanisms including the biosynthesis and release of a blend of herbivore-induced plant volatiles (HIPVs) (Arimura et al. 2009). These compounds can function in indirect plant defence by attracting natural enemies of herbivores (Fontana et al. 2011) or directly to repel insects such as aphids and caterpillars (Aharoni et al. 2003; Beale et al. 2006; Irmisch et al. 2014). Among the HIPVs, terpenes – especially mono-, sesqui- and homoterpenes (degradation products) – have been frequently reported. In maize, plants produce (*E*)-β-farnesene, (*E*)-nerolidol, and (*E*,*E*)-farnesol after herbivore damage (Schnee et al. 2002). In Arabidopsis, plants genetically engineered to produce linalool have been shown to repel aphids (Aharoni et al. 2003) whilst in tea plants, the sesquiterpene α-farnesene was produced after leaves were infested by *Adoxophyes honmai* (Dong et al. 2011). Hormones like jasmonic acid (JA) have been shown to be involved in the regulation of terpene biosynthesis.

Monoterpene and sesquiterpene substrates geranyl- and farnesyl diphosphate (GDP and FDP respectively) are biosynthesised by condensing one dimethylallyl diphosphate (DMAPP) molecule with one or more isoprenoid molecules, isopentenyl diphosphate (IDP) (McGarvey and Croteau 1995) by the action of prenyl transferases. In plants, the two main substrate pathways for volatile terpene production are spatially separated in two compartments. The methylerythritol phosphate (MEP) pathway and the mevalonic acid (MVA) pathway, act in the plastids (Banerjee and Sharkey 2014) and cytosol/peroxisomes (Lichtenthaler 1999), respectively. However, cross-talk between the MEP and MVA pathway also exists (Hemmerlin et al. 2003) and the MEP pathway has also been reported to serve for both monoterpene and sesquiterpene biosynthesis (Dudareva et al. 2005). The final step of volatile terpene biosynthesis is catalysed by terpene synthase (TPS) enzymes, to form monoterpenes and sesquiterpenes (Bohlmann et al. 1998). Terpene skeletons can be further modified by various enzymes, for example, the homoterpene compounds DMNT and TMTT (4,8,12-trimethyltrideca-1,3,7,11-tetraene) are derived from the oxidative degradation of the sesquiterpene (*E*)-nerolidol and the diterpene (*E*,*E*)-geranyllinalool, respectively, by cytochrome P450 monooxygenases (CYPs) (Lee et al. 2010). In some plants, UDP-glycosyltransferases can catalyse the transfer of an activated nucleotide sugar to acceptor aglycones to sequester terpenes as non-volatile glycosides e.g. Bönisch et al. (2014).

TPS enzymes contain conserved sequence motifs such as the aspartate-rich DDXXD motif and NSE/DTE motif in their C-terminal catalytic domain which plays functional roles in substrate binding and enzyme catalysis (Aubourg et al. 2002; Bohlmann et al. 1998; Chen et al. 2011; Christianson 2006; Zhou and Peters 2009). They can also contain an RR(X)8W motif at the N-terminal involved in the initiation of the isomerisation cyclisation reaction (Williams et al. 1998) or in stabilising the protein through electrostatic interactions (Hyatt et al. 2007). Many individual volatile TPS genes from different species have been isolated from specific tissues e.g. flowers or fruit, or in response to different stimuli e.g. mechanical wounding or herbivory. However, the complete genomic complement of volatile TPS genes has been identified and characterised in a relatively small number of plant species e.g. grape (Martin et al. 2010), apple (Nieuwenhuizen et al. 2013) and *Lathyrus odoratus* (Bao et al. 2020). Analysis of the full complement of volatile TPS genes allows functional redundancy of TPS genes and the overlapping roles of terpenes in different tissues to be considered.

In this study, our aim was to identify, functionally characterise and better understand the biological functions of the full set of TPS enzymes in kiwifruit. In Red5 kiwifruit we analyse terpenes in different tissues and during different stages of development, then use the high-quality *A. chinensis* var. *chinensis* Red5 reference genome (Pilkington et al. 2018) to identify the full complement of TPS genes in the kiwifruit genome responsible for volatile terpene biosynthesis. In the key commercial kiwifruit cultivars ‘Hort16A’ and ‘Hayward’ we report the response of TPS gene to MeJA and to herbivory by the economically important insect pest brown-headed leaf roller. Our results further revealed that the small TPS gene family in kiwifruit shows surprisingly high functional redundancy and a subset of terpene synthases likely fulfil overlapping functions in fruit flavour, floral bouquet and plant defence.

## Results

### Temporal and spatial distribution of terpenes in kiwifruit

**Fig. 1:**
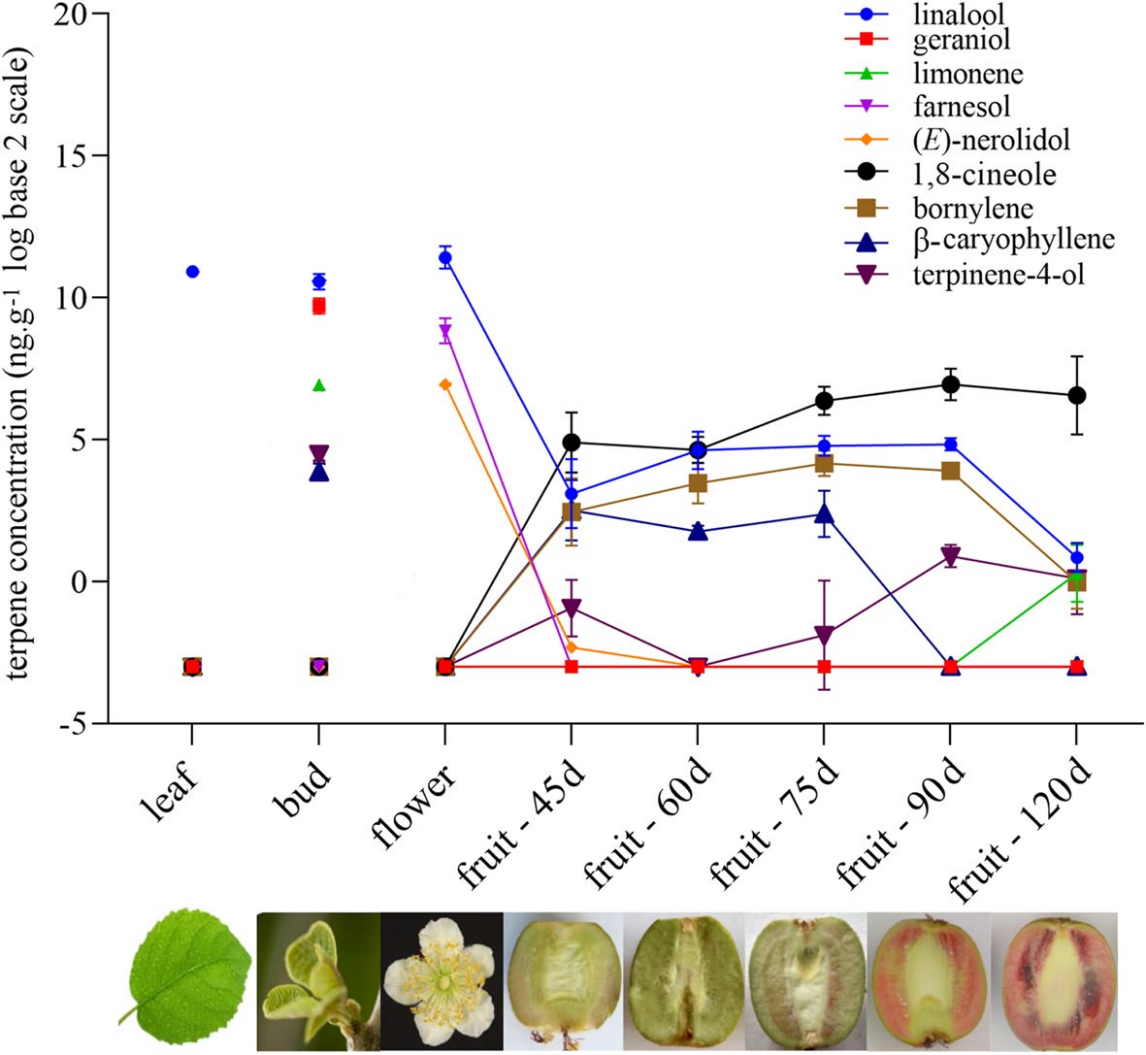
Terpene volatiles in Red5 tissues and developing fruit. Terpene volatiles were collected by SPME and analysed by GC–MS from developing fruit peel at 45–120 days (d) after anthesis. Flowers were collected when fully open and included petals, stamens and ovaries. Buds were harvested at less than 2 cm. Leaves were sampled at the mature, full-expanded stage. Terpene concentrations are expressed in log base 2, undetected terpenes were given values of 0.01 ng∙g^−1^ FW. Data are means ± SE (*n* = 3). Table S1 shows data for all terpene volatiles detected

Previous studies have shown that significant differences exist in the abundance and composition of terpenes in flowers and fruit across various *Actinidia* species (Crowhurst et al. 2008; Nieuwenhuizen et al. 2015) and among different cultivars and accessions within the same species (Wang et al. 2021). The availability of a high-quality reference genome for diploid *A. chinensis var. chinensis* Red5 (Pilkington et al. 2018) has made this variety a key tool in gene discovery efforts. Therefore, a detailed analysis of terpene composition in a range of Red5 tissues and fruit developmental stages was undertaken by GC–MS after solid phase micro extraction (SPME) (Fig. 1, Table S1). In whole flowers, linalool was highly abundant (2800 ng∙g^−1^ fresh weight – FW) along with farnesol (470 ng∙g^−1^ FW) and (*E*)-nerolidol (123 ng∙g^−1^ FW). These compounds were also the major compounds detected in flowers of *A. chinensis* ‘Hort16A’ (Green et al. 2012), but the lilac compounds present in *A. arguta* flowers were not observed (Chen et al. 2010). In buds, linalool, geraniol and limonene were the major compounds detected. In fruit, 1,8-cineole was the most abundant terpene detected (max. 130 ng∙g^−1^ FW), confirming the results from Zeng et al. (2020). A range of other terpenes including β-caryophyllene and bornylene were detected in developing fruit, but generally at relatively low concentration (< 30 ng∙g^−1^ FW). Linalool was the major compound detected in young expanding leaves.

### Identification of TPS genes in the Red5 genome

The Red5 genome (Pilkington et al. 2018) and the corresponding annotated gene models (designated ‘-*R5’*) were interrogated using BLASTP with known TPS sequences from kiwifruit, Arabidopsis, and tomato as queries. Twenty-two unique TPS gene models were identified (Table S2A) and systematically named as *AcTPS1-R5*–*AcTPS22-R5*. Eleven TPS genes and alleles previously reported and functionally characterised in various kiwifruit species (Table S2B) were identified in the Red5 genome (Table 1): *AcTPS8-R5* and *AcTPS11-R5* correspond to *AdAFS1* (α-farnesene synthase) and *AdGDS* (germacrene-D synthase) from *A. deliciosa* (Nieuwenhuizen et al. 2009); *AcTPS13-R5* corresponds to linalool synthases *AaLS1* and *ApLS1* from *A. arguta* and *A. polygama* (Chen et al. 2010) and *AcTPS14-R5* corresponds to *AcNES1* (nerolidol synthase) previously isolated from *A. chinensis* ‘Hort16A’ (Green et al. 2012). Five Red5 gene models located on chromosome 29 (*AcTPS16-R5*–*AcTPS-20-R5*) have been resolved to four functional TPS genes (*AcTPS1a*–*d*) in Red5 (Zeng et al. 2020) and are likely alleles/orthologues of *AcTPS1* and *AaTPS1* from ‘Hort16A’ and *A. arguta* (Nieuwenhuizen et al. 2015). Five Red5 gene models were truncated and likely represent non-functional pseudogenes (*AcTPS2-, 4-, 6-, 12-, 22-R5*, Table S2A).

**Table 1:** Properties of fifteen full-length AcTPS genes identified in the Red5 genome

Red5 Gene model	Classification	Closest homolog	Id (%)	TPS clade	Chromosome	Protein length	Full length Red5 TPS	Deduced products
Acc09580	AcTPS1-R5	AdAFS1	72.8	e/f	8	776	AcNES3	(*E*)-nerolidol
Acc13525	AcTPS3-R5	CsRLIS	66.5	g	12	546	AcNES2	(*E*)-nerolidol
Acc13742	AcTPS5-R5	AdGDS	76.5	a	12	561	AcBCS	β-caryophyllene
Acc15182	AcTPS7-R5	CsCPS	73.5	c	13	824	AcCPS	ent-copalyl diphosphate
Acc17359	AcTPS8-R5	AdAFS1	96.3	e/f	15	768	AcAFS1	α-farnesene
Acc19057/ Acc19058	AcTPS9-R5/ AcTPS10-R5	CsRLIS	66.2	g	17	579	AcLIS/NES	linalool/(*E*)-nerolidol
Acc19649	AcTPS11-R5	AdGDS	88.9	a	17	565	AcGDS	germacrene-D
Acc20592	AcTPS13-R5	AaLS1	90.9	g	18	574	AcLS1	linalool
Acc25053	AcTPS14-R5	AcNES1	97.7	g	22	573	AcNES1	(*E*)-nerolidol
Acc26061	AcTPS15-R5	OeGES1	57.7	g	23	575	AcGES	geraniol
Acc32631	AcTPS16-R5	AcTPS1a	*	b	29	603	AcTPS1a	sabinene
Acc32632	AcTPS17-R5	AcTPS1b	*	b	29	603	AcTPS1b	1,8-cineole
Acc32633	AcTPS18-R5	AcTPS1	*	b	29	603	AcTPS1c	geraniol
Acc32635	AcTPS19-R5	AcTPS1d	*	b	29	605	AcTPS1d	diterpene
Acc32636	AcTPS20-R5	AaTPS1, AcTPS1c	*	b	29	604/603	**	α-terpinolene/ β-myrcene
Acc33493	AcTPS21-R5	CsKS1	76.7	e/f	24	793	AcEKS	ent-kaurene

Red5 Gene model: The official IASMA (Instituto Agrario San Michele all’Adige) ‘gene model’ ID in the Red5 kiwifruit genome from the Genome Database for http://plants.ensembl.org/Actinidia_chinensis/Info/Index. Classification: AcTPS-R5 nomenclature for the fifteen full-length terpene synthase (TPS) gene models identified in the Red5 genome. Closest homolog: Best BLASTp hit with a defined function to each AcTPS in the GenBank non-redundant (NR) protein database. Id (%). Percent amino acid identity to the closest homolog. TPS clade a–h: classification of the TPS gene models according to phylogenetic and functional studies (Chen et al. 2011). Chromosome: Chromosomal location of each AcTPS in the Red5 genome. Protein length: Predicted amino acid number of the AcTPS open reading frame. Red5 AcTPS functional nomenclature and main terpene products deduced. Ad = *A. chinensis* var. *deliciosa*, Ac = *A. chinensis* var. *chinensis*, Aa = *A. arguta*, Cs = *Camellia sinensis*, Oe = *Olea europaea*. Full gene names are given in Table S3.

^*^ The five gene models Acc32631–6 are part of a complex locus in the Red5 genome (Zeng et al. 2020). None of the gene models corresponded 100% to the four genes *AcTPS1a*–*d* amplified from Red5 cDNA.

^**^ AaTPS1, AcTPS1 are likely allelic variants at the AcTPS1 locus that produce α-terpinolene and ß-myrcene respectively (Nieuwenhuizen et al. 2015)

Of the remaining eight gene models, two represent putative full-length genes encoding ent-copalyl diphosphate (*AcCPS*) and ent-kaurene synthases (*AcEKS*) involved in the biosynthesis of the non-volatile gibberellins (Keeling et al. 2009). Three gene models (*AcTPS1-R5*, *3-R5* and *5-R5*) were full-length and showed highest homology to TPS genes encoding α-farnesene, linalool and germacrene-D synthases (Table 1). Primers designed to these three gene models amplified TPS proteins of 776, 546 and 561 amino acid residues respectively (Table 1). Two gene models (*AcTPS9-R5* and *AcTPS10-R5*) appeared to be incorrectly annotated as separate genes. A full-length TPS gene with homology to *CsRLIS* (*Camellia sinensis* (*R*)-linalool synthase, QNI69163.1) was amplified using primers to the N-terminus of *AcTPS9-R5* combined with the C-terminus of *AcTPS10-R5* (Figure S1) resulting in a 579 amino acid (aa) open reading frame (ORF). *AcTPS15-R5* encodes a truncated gene model of 1410 bp (470 aa). The N-terminus of *AcTPS15-R5* was extended by studying RNAseq reads upstream of the existing gene model’s start codon (Figure S1). The full-length *AcTPS15-R5* encodes a protein of 575 aa. Alignment of the predicted amino acids of Red5 TPS genes showed several conserved motifs (Figure S2). *AcTPS3-R5, AcTPS5-R5* and *AcTPS9/10-R5* contained both the RRX(8)W, DDXXD and NSE/DTE motifs, while *AcTPS1-R5* and *AcTPS15-R5* only contained the DDXXD and NSE/DTE motif (Figure S2).

### Phylogenetic analysis and chromosomal location of putative AcTPS genes

**Fig. 2:**
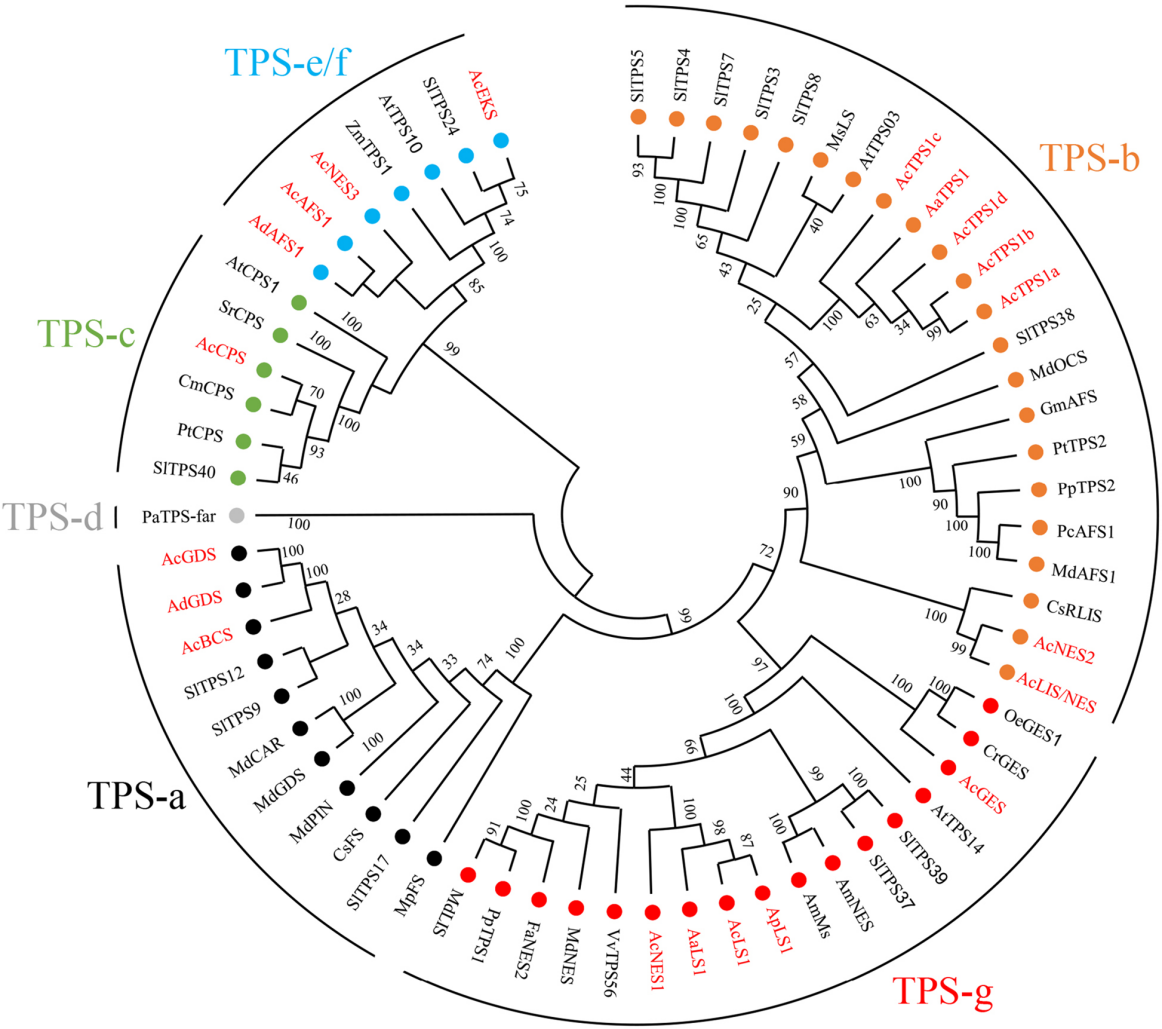
Phylogenetic analysis of the Red5 TPS gene family. Predicted full-length and functional TPS genes identified in the Red5 genome and previously characterised *Actinidia* TPS genes were aligned with published full-length TPS genes using ClustalW in Geneious (v. R10). The phylogenetic tree was constructed using the neighbour-joining method in MEGA (v7.0.14). Bootstrap values are shown as a percentage of 1000 replicates. *Actinidia* TPS genes are highlighted in red. TPS clades are based on (Chen et al. 2011). Full gene names and GenBank accession numbers are provided in Table S3

To investigate the evolutionarily relationships of *Actinidia* TPS genes, a phylogenetic tree was generated comparing previously characterised functional kiwifruit TPS genes, the fifteen full-length AcTPS-R5 genes and selected TPS from other species (Fig. 2; Table S3). The results showed that all the *Actinidia* TPS proteins clustered into angiosperm-specific clades (Fig. 2). The TPS-b and TPS-g subclades include the highest number of AcTPS genes, with five and eight members respectively and include members that function as either mono- or sesquiterpene synthases. *AcTPS5-R5* clusters with other TPS-a subfamily members including the previously characterised *AdGDS* (germacrene-D synthase) (Nieuwenhuizen et al. 2009), whilst *AcTPS1-R5* clusters with *AdAFS1* (α-farnesene synthase) into TPS-e/f.

**Fig. 3:**
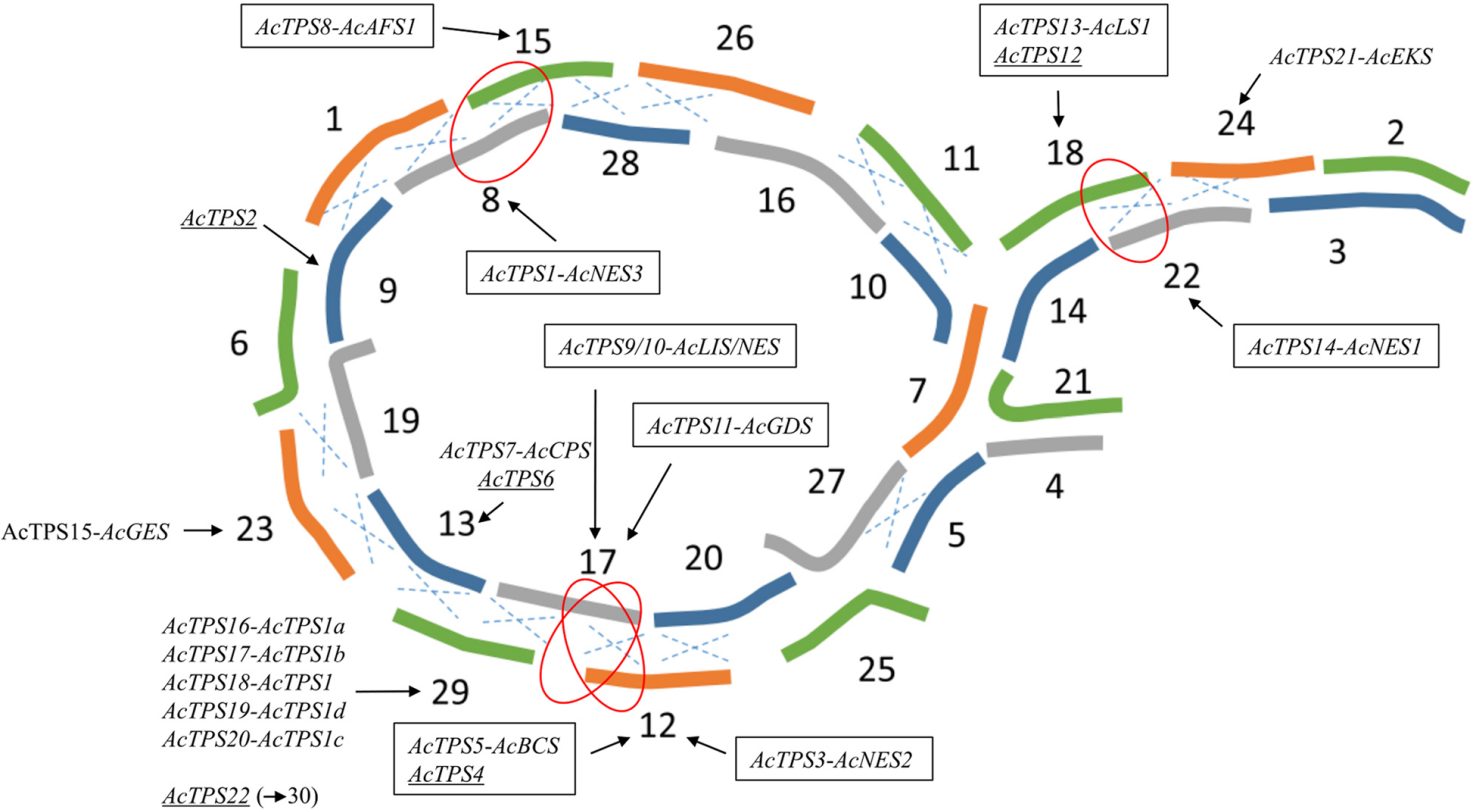
Location of full-length AcTPS and pseudogenes on the 29 chromosomes in Red5. A dashed ‘x’ shows a region where translocations have occurred at the centromeric regions. Chromosomes not aligned with a dashed ‘x’ show regions of homology due to other proposed chromosomal rearrangements (Pilkington et al. 2018). Red circles highlight regions where pairs of TPS genes (boxed) on homeologous chromosomes are located. Pseudogenes are underlined. Figure adapted from (Pilkington et al. 2018)

In order to gain better insight into the evolution of TPS in kiwifruit, the chromosomal location was determined for each of the Red5 TPS genes (Fig. 3). The largest number of TPS genes occurred on chromosome 29 (*n* = 6) which contains the tandemly repeated *AcTPS1a*–*d* cluster previously characterised by Zeng et al. (2020), which suggests the occurrence of multiple duplication and recombination events may have occurred on chromosome 29. As the diploid kiwifruit genome is the result of several ancient duplication events (Pilkington et al. 2018), homeologous pairs of duplicate of TPS can also be expected. Two pairs of TPS genes were identified on homeologous chromosomes 12 and 17: *AcTPS3-R5::AcTPS9/10-R5* and *AcTPS5-R5*::*AcTPS11-R5*. *AcTPS1-R5*::*AcTPS8-R5* are located on homeologous chromosomes 8 and 15 and *AcTPS13-R5*::*AcTPS14-R5* are located on homeologous chromosomes 18 and 22 (Fig. 3). A number of the TPS pseudogenes identified in the Red5 genome appear to be non-functional paralogues of neighbouring genes e.g. *AcTPS4-R5* and *AcTPS5-R5* on chromosome 12 and *AcTPS12-R5* and *AcTPS13-R5* on chromosome 18*.*

### Tempo-spatial expression patterns of AcTPS genes in different organs

**Fig. 4:**
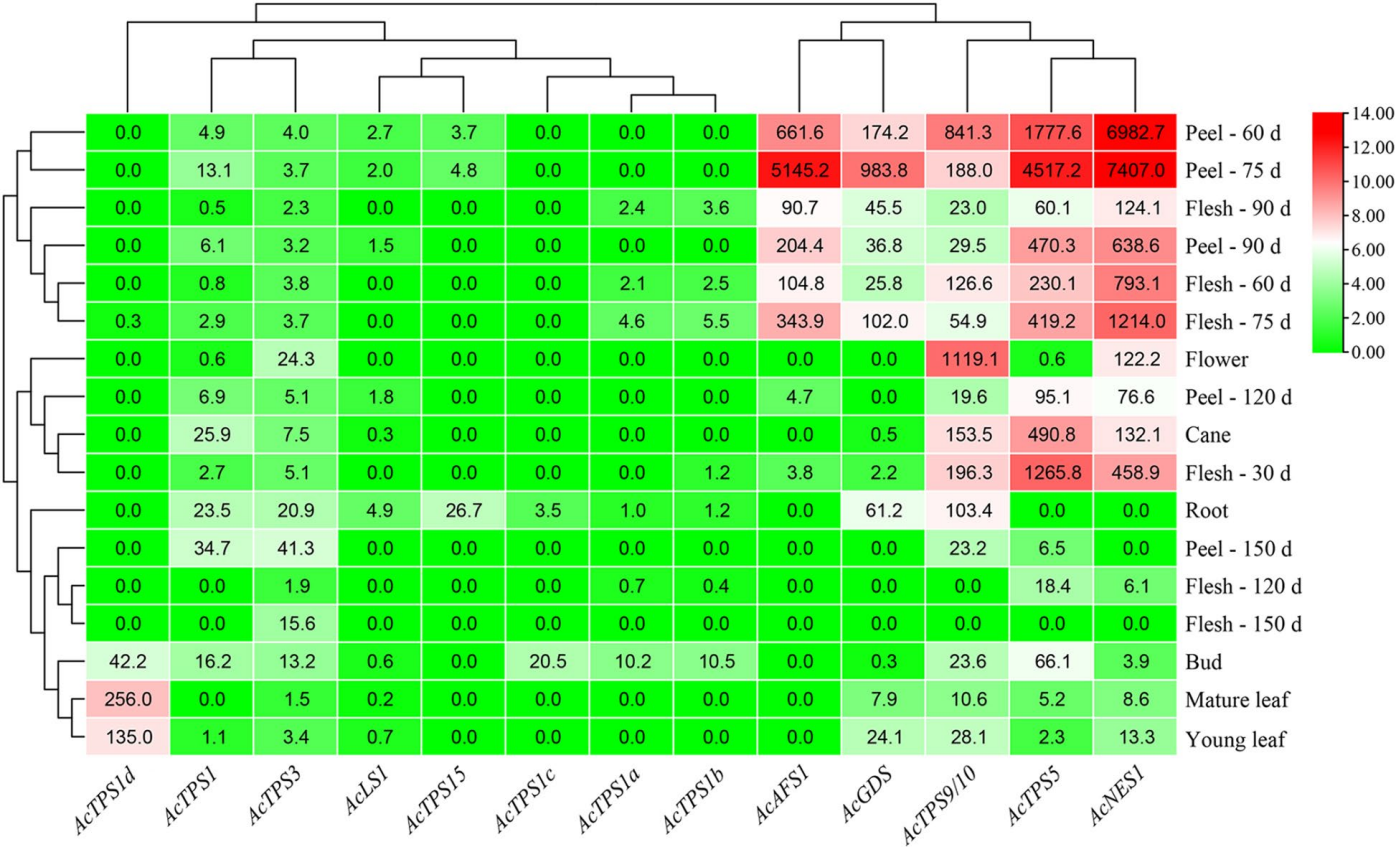
Analysis of volatile terpene synthase gene expression in different kiwifruit tissues and time points. Red5 leaf, flower and bud samples for qRT-PCR correspond to those sampled in Fig. 1. Peel and flesh samples were obtained from developing fruit at 30–150 days (d) after anthesis. The gene-specific primers used are given in Table S7. See Table 1 for full TPS gene details. Expression is given relative to the young leaf sample of *AcTPS1*. 0.0: undetectable expression. Data are means of relative expression (*n* = 3). Hierarchical clustering was performed using TBtools v 1.0985. The scale bar and colouring was built based on the gene expression values in log base 2

To determine the temporal changes and tissue distribution of TPS transcripts, expression was measured in a total of 17 kiwifruit tissues (i.e. buds, young leaves, mature leaves, cane, flowers, fruit and roots). Relative quantification real-time PCR (qRT-PCR) was used with gene-specific primers designed to all thirteen TPS genes likely involved in producing volatile terpene products (i.e. excluding *AcCPS* and *AcEKS* involved in non-volatile gibberellin biosynthesis). The expression levels are presented as a heat map in Fig. 4. Distinct tempo-spatial expression patterns were observed for all TPS genes. Several genes such as *AcGDS*, *AcAFS1* and *AcNES1* were expressed in the majority of tested samples, apart from very low levels of expression in fruit flesh and peel at 150 d. In contrast, the expression of other genes was undetectable in most of the tested tissues (e.g. *AcTPS1c*, *d*). Of the newly identified volatile TPS genes, *AcTPS15-R5* was predominantly expressed in young peel samples and root. *AcTPS5-R5* was expressed strongly in developing fruit and bud tissues. *AcTPS1-R5* and *AcTPS3-R5* showed similar expression patterns with relatively high expression in roots, bud and ripe fruit (150 d) peel tissues. *AcTPS9/10-R5* showed the highest expression in flower and 60 d fruit peel, but was expressed at lower levels in other organs such as mature fruit and leaves.

### Functional characterisation of the five new volatile-related AcTPS genes by transient over-expression in planta

**Fig. 5:**
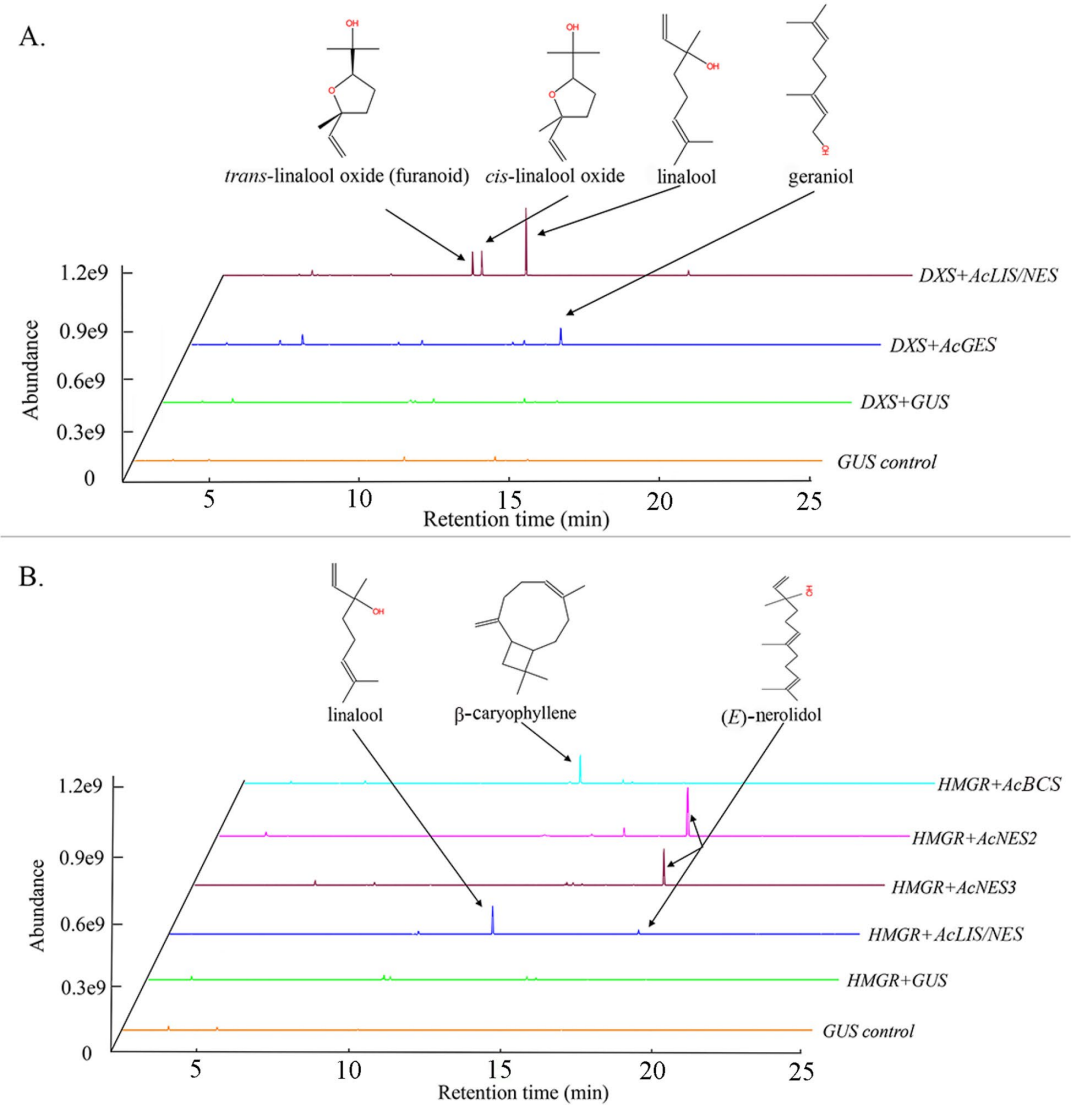
Volatile terpenes produced by transient over-expression of AcTPS genes in planta. *N. benthamiana* leaves were infiltrated with *Agrobacterium* suspensions containing pHEX2_LIS/NES, pHEX2_AcGES, pHEX2_AcBCS, pHEX2_AcNES2, pHEX2_NES3 or the negative control pHEX2_GUS. Leaves were infiltrated in combination with pHEX2-AcDXS (**A**) or pEAQ-tHMGR-2ABCCP1 (**B**) to up-regulate flux through the MEP and mevalonate pathways, respectively. Volatiles were collected by SPME and analysed by GC–MS 7 d post infiltration. Experiments were performed in triplicate, and a single representative trace is shown (based on the single ion *m/z* 93). The concentrations of all terpene volatiles measured after inoculation are presented in Table S4

Of the thirteen AcTPS genes from Red5 likely involved in producing volatile terpene products, *AcTPS1a-d* have previously been biochemically characterised (Zeng et al. 2020) and four (*AcAFS1*, *AcGDS*, *AcLS1*, *AcNES1*) were assigned likely functions based on high sequence similarity (> 90%) to genes previously assigned activities in other *Actinidia* species (Table 1). Transient over-expression in *Nicotiana benthamiana* was used to determine the functional characteristics of the five new volatile-related kiwifruit TPS genes in planta. Binary vector constructs for each TPS were co-infiltrated into *N. benthamiana* leaves together with pHEX2_AcDXS (1-deoxy-D-xylulose-5-phosphate synthase) to increase monoterpene substrate production by the MEP pathway or with pEAQ-tHMGR-2A-BCCP1 (3-hydroxy-3-methylglutaryl-coenzymeA reductase) to increase sesquiterpene production by the mevalonate pathway (Zeng et al. 2020). The results (Fig. 5A/B; Table S4) showed that *AcTPS15-R5* mainly catalysed the formation of the monoterpene geraniol and therefore it was named *A. chinensis* geraniol synthase (*AcGES*). *AcTPS5-R5* (*AcBCS*) produced the sesquiterpene β-caryophyllene. *AcTPS3-R5* (*AcNES2*) and *AcTPS1-R5* (*AcNES3*) both produced mainly (*E*)-nerolidol, while *AcTPS9/10-R5* (*AcLIS/NES*) made mostly linalool and its oxidised derivatives plus a small amount of nerolidol (Fig. 5A/B; Table S4).

The sesquiterpene synthases *AcNES2*, *AcNES3* and *AcBCS* showed between 2–20 fold increases in sesquiterpene production by increasing the mevalonate-derived substrate pool by up-regulating HMGR (Table S4). *AcGES* produced ~ tenfold higher levels of geraniol in combination with DXS (Table S4). *AcLIS/NES* showed a ~ 70-fold increase in (*E*)-nerolidol production in combination with HMGR, but still produced larger absolute amounts of linalool (> 4-fold) and derivatives in the presence of HMGR. When combined with DXS, a 20-fold increase in linalool and derivatives was observed. Overall, (*E*)-nerolidol levels with HMGR were > 100-fold lower than linalool with DXS. These results suggest that *AcLIS/NES* is primarily a monoterpene synthase in planta supported by the MEP substrate pathway and produces much smaller amounts of (*E*)-nerolidol supported by the mevalonate pathway (Fig. 5A/B; Table S4).

### Enzymatic characterisation of the five new volatile-related kiwifruit AcTPS genes in vitro

**Fig. 6:**
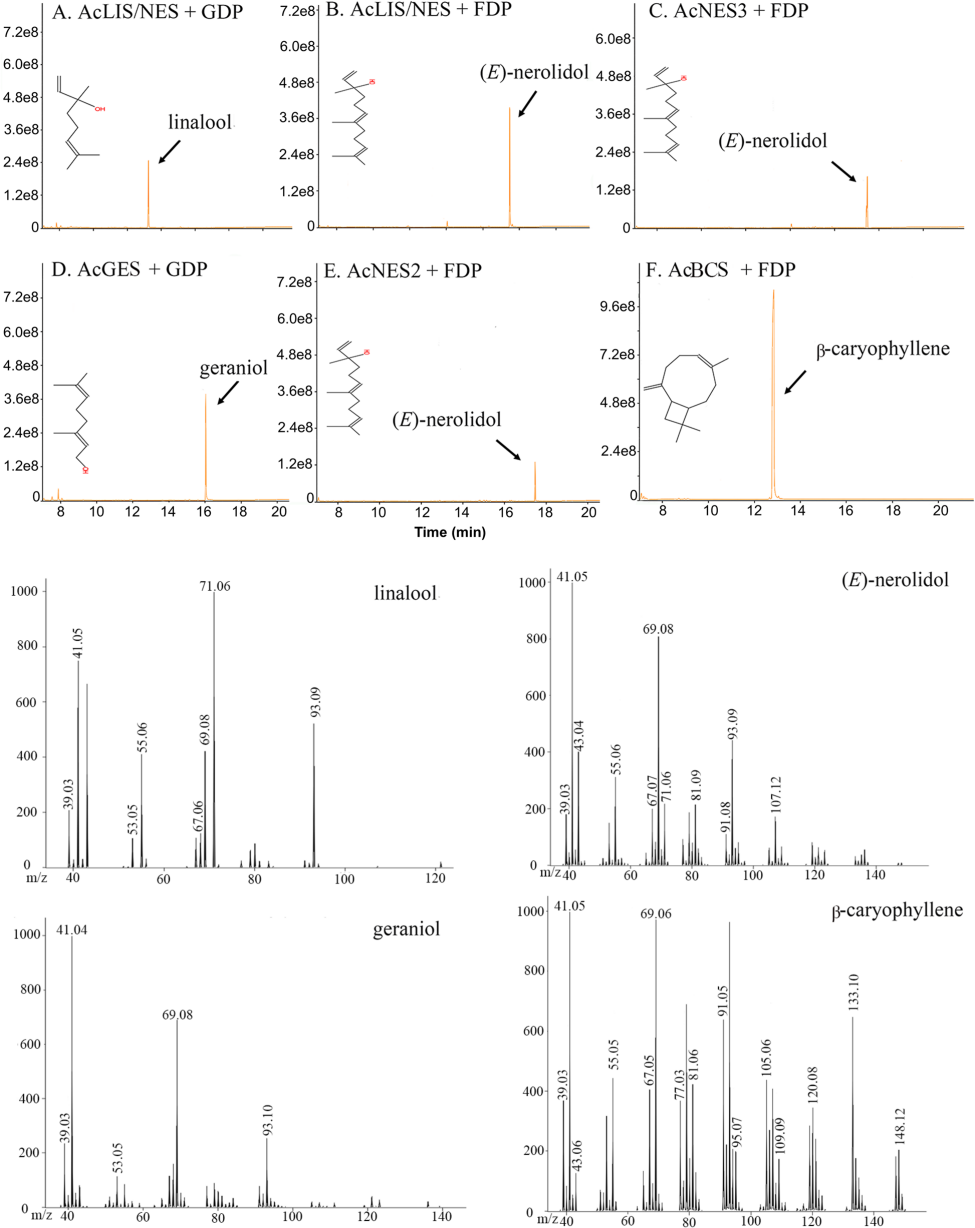
Volatile terpenes produced by over-expression of AcTPS genes in *E. coli*. Recombinant enzymes were purified by Ni^2+^ affinity and gel filtration chromatography. Terpene volatiles were collected by SPME and analysed by GC–MS in triplicate. **A** AcLIS/NES + GDP as substrate; **B** AcLIS/NES + FDP; **C** AcNES3 + FDP; **D** AcGES + GDP; **E** AcNES2 + FDP; **F** AcBCS + FDP. The X-axis represents the retention time of the peak outflow and the Y-axis represents the relative intensity of the chromatographic peak. The lower panel shows the mass-to-charge ratios (*m/z*). The concentrations of all terpene volatiles measured are detailed in Table S5

To determine the activity of the five new volatile-related TPS kiwifruit TPS genes in vitro, recombinant HIS-tagged proteins were expressed in the *Escherichia coli* and proteins were purified by Ni^2+^ affinity purification. The products formed using GDP or FDP as substrates were analysed by SPME GC–MS. The in vitro results for the five active recombinant TPS enzymes (Fig. 6; Table S5) were very similar to those obtained for the transient in planta expression analysis.

AcLIS/NES produced the monoterpene linalool (87%) and the sesquiterpenes (*E*)-nerolidol (64%) as major products from GDP and FDP respectively (Fig. 6; Table S5). The reaction products formed by AcNES2 and AcNES3 with FDP were (*E*)-nerolidol (94% and 83% respectively). AcGES and AcBCS produced geraniol (81%) and β-caryophyllene (100%) in the presence of GDP or FDP respectively (Fig. 6; Table S5).

Enzyme kinetic analysis (Table 2) of the purified recombinant AcTPS proteins showed that the monoterpene synthase AcGES showed higher affinity towards GDP compared to AcLIS/NES (0.8 vs 31 µM), whilst AcBCS showed the highest affinity towards FDP (1.5 µM) with AcNES3 the lowest (85 µM). Unexpectedly, the dual function enzyme AcLIS/NES showed a higher *Km* with GDP (31 µM) versus FDP (7 µM) and also a lower catalytic efficiency (*Kcat/Km*) value with GDP versus FDP (0.00678 vs. 0.1103 s^−1^∙mM^−1^, respectively). In planta, the enzyme mainly produced linalool. Comparing the catalytic efficiency of the three (*E*)-nerolidol biosynthesis related proteins (AcLIS/NES, AcNES2 and AcNES3) indicated that AcLIS/NES showed the highest efficiency with FDP (0.1103 s^−1^∙mM^−1^) and the highest turnover rate (*Kcat* of 0.00082 s^−1^).

**Table 2:** Kinetic properties of recombinant TPS enzymes

	**K**_**m**_** (μM)**	**V**_**max**_** (nM∙min**^**−1**^**)**	**K**_**cat**_** (s**^**−1**^**)**	**K**_**cat**_**/K**_**m**_** (s**^**−1**^**∙mM**^**−1**^**)**
**AcBCS**				
FDP	1.54 ± 0.54	13.00 ± 3.10	0.0087 ± 0.0020	5.87 ± 1.10
**AcNES2**				
FDP	5.55 ± 0.73	0.30 ± 0.03	1.89E-05 ± 2.07E-06	3.48E-03 ± 8.73E-04
**AcLIS/NES**				
GDP	30.95 ± 3.81	3.38 ± 0.22	0.00021 ± 0.0001	0.00678 ± 0.0011
**AcLIS/NES**				
FDP	7.43 ± 1.25	4.33 ± 0.12	0.00082 ± 0.0002	0.1103 ± 0.025
**AcNES3**				
FDP	84.69 ± 18.46	1.80 ± 0.01	0.00026 ± 0.0001	0.00307 ± 0.00046
**AcGES**				
GDP	0.76 ± 0.21	3.36 ± 0.23	0.00143 ± 0.0003	1.88 ± 0.65

Kinetic parameters of purified recombinant enzymes were determined in 50 mM Bis–Tris propane buffer pH 7.5. Parameters for FDP (0–50 μM) were obtained in the presence of 10 mM MgCl_2_. All values represent mean ± SE, *n* = 3. K_m_, Michaelis constant; V_max_, maximum velocity; K_cat_, turnover

### Subcellular localisation of AcTPS genes

**Fig. 7:**
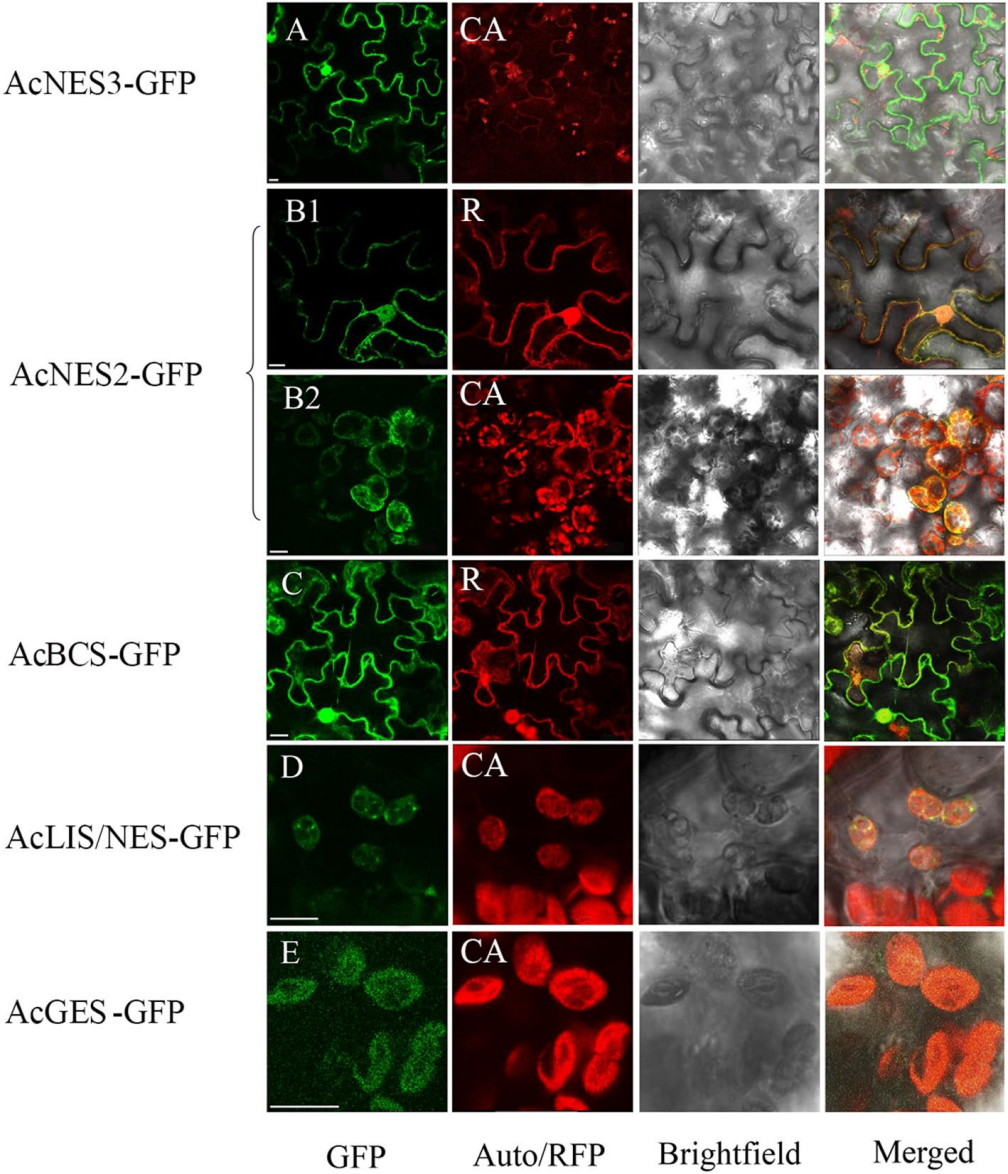
Subcellular localisation of AcTPS in protoplasts. AcTPS-GFP translational fusion constructs were transiently expressed in *N. benthamiana* and analysed by confocal laser-scanning microscopy. GFP: GFP fluorescence; CA: chlorophyll auto-fluorescence from chloroplasts; R: fluorescence of red fluorescence protein from the vector (pMDC43) localised to the cytoplasm; brightfield: light microscopy images of intact mesophyll protoplasts; merged: visible and fluorescence signals (GFP + CA or GFP + R) combined. (A) AcNES3-GFP targeted to the cytoplasm. (B1) AcNES2-GFP targeted to the cytoplasm and (B2) to the chloroplast. (C) AcBCS-GFP targeted to the cytoplasm. (D) AcLIS/NES-GFP targeted to the chloroplast. (E) AcGES-GFP targeted to the chloroplast. Scale bars = 10 µm

To determine the subcellular localisation of the five new volatile-related AcTPS genes, full-length ORFs were fused at the C-terminus to the GFP reporter gene and infiltrated into *N. benthamiana* leaves. Transient expression of GFP in protoplasts was then analysed by confocal laser scanning microscopy. Strong GFP fluorescence signals for AcNES3-GFP were detected in the cytoplasm (Fig. 7A, panel 1), whilst non-overlapping punctate red spots were observed in the corresponding chlorophyll autofluorescence (CA) image (Fig. 7A, panel 2).

For AcNES2-GFP, relatively weak GFP fluorescence signals were observed in the cytoplasm where ChloroP (Emanuelsson et al. 2000) predicted the gene to be localised (Fig. 7B1), but also some in chloroplasts (Fig. 7B2). GFP fluorescence signals for the sesquiterpene synthase AcBCS-GFP construct were detected in the cytoplasm (Fig. 7C, panel 1) coincident with fluorescence of RFP from the vector (pMDC43) localised to the cytoplasm (Fig. 7C, panel 2). For AcLIS/NES-GFP the GFP fluorescence signal was localised to the chloroplasts, suggesting that the primary function of *AcLIS/NES *in planta would be as a monoterpene (linalool) synthase utilising the chloroplastic pool of GDP (Fig. 7D) consistent with the in planta transient expression data (Fig. 5). The merged GFP signal from AcGES-GFP and chloroplast autofluorescence indicated that the monoterpene synthase *AcGES* was localised to the chloroplast (Fig. 7E). No GFP or RFP fluorescence was detected in untransfected protoplasts.

### Terpene production and AcTPS expression in leaves are up-regulated by MeJA treatment

**Fig. 8:**
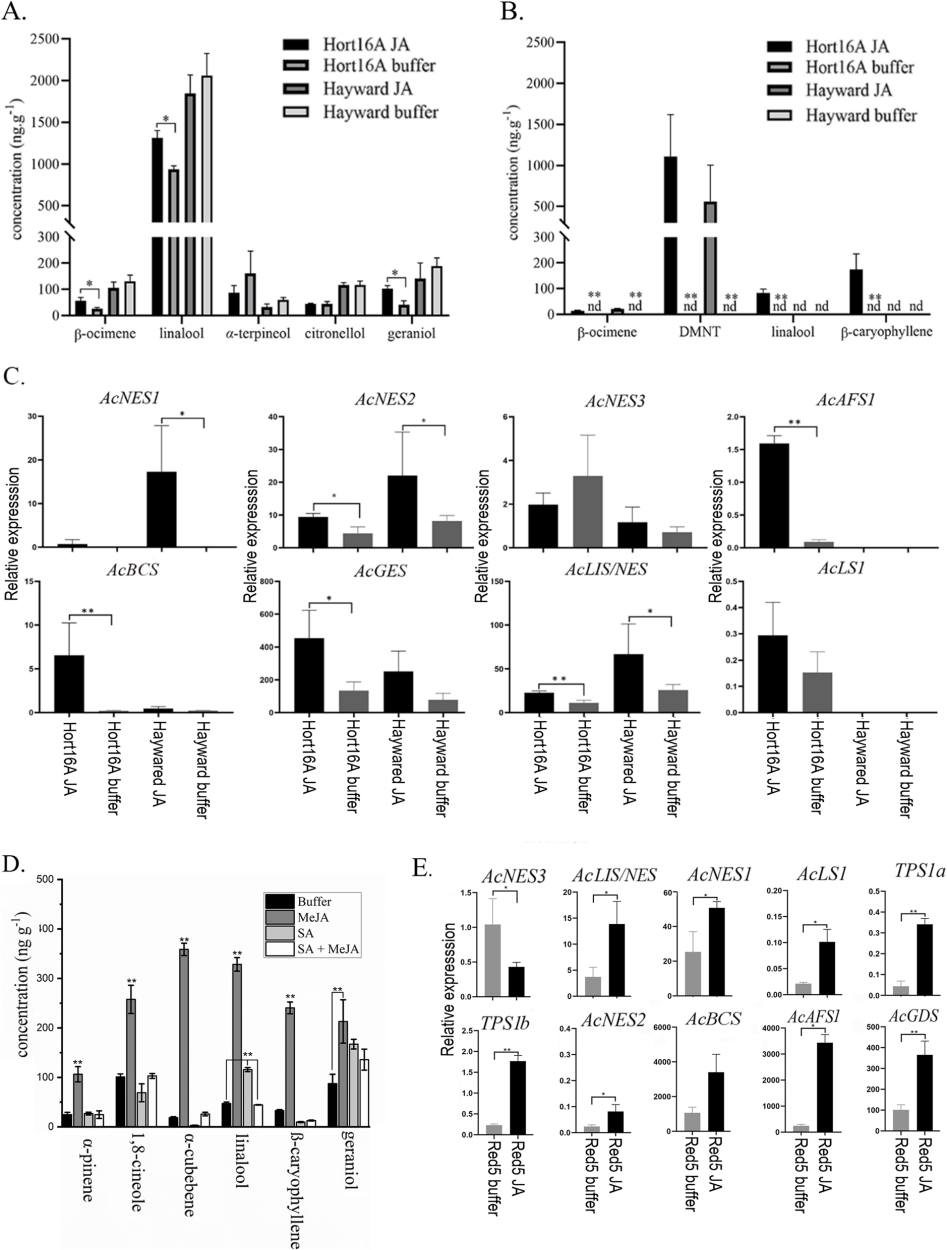
Terpene production and *AcTPS* gene expression in tissue-cultured leaves and young fruit after hormone treatment. **A** Terpene accumulation in tissue-cultured leaves of kiwifruit ‘Hort16A’ and ‘Hayward’ after MeJA (JA) treatment. Leaf tissues were ground to powder and terpenes analysed by SPME GC–MS. **B** Terpene emission from tissue-cultured leaves of kiwifruit after MeJA treatment. Terpenes were collected by Tenax TA for 5 d and analysed by GC–MS. **C** Expression of all full-length AcTPS genes affected by MeJA treatment in tissue-cultured leaves. Gene expression was determined by qRT-PCR. **D** Terpenes accumulating in the tissue of 45 d old Red5 fruit after treatment with MeJA, SA and SA + MeJA. Fruit tissues were ground to powder and terpenes analysed by SPME GC–MS Compounds detected in fruit at > 100 ng∙g^−1^ are shown. **E** Expression of all full-length *AcTPS* genes affected by MeJA treatment of young fruit. Gene expression was determined by qRT-PCR. Data are means ± SE (*n* = 3). The Student’s t-test was used to test for statistical significance. Asterisks indicate a significant difference (*P < 0.05, **P < 0.01) between MeJA treated and untreated samples

Defence-related terpene emissions from plants are commonly mediated by JA-dependent signalling pathways and those of other phytohormones (Ponzio et al. 2013). To investigate this process during vegetative development in kiwifruit leaves, tissue-cultured plants of two commercially available kiwifruit cultivars ‘Hayward’ and ‘Hort16A’ were treated with MeJA. Considerable knowledge on the susceptibility of both cultivars to pathogens and insects is available. Both cultivars are routinely propagated commercially in tissue culture, providing access to plants for replicated analyses. Terpene accumulation inside the leaf tissue, as well as the emission/release of monoterpenes into the headspace, were both measured by GC–MS. High levels of linalool (and derivative oxides) and lower levels of other terpenes such as β-ocimene, α-terpineol and geraniol (Fig. 8A, Table S6A) were present in the tissue of both control and treated leaves. In ‘Hort16A’, significantly higher levels of linalool, geraniol and β-ocimene accumulated after MeJA treatment compared to control leaves, while the levels were unchanged in ‘Hayward’. Different patterns of volatile release were also observed in the headspace samples (Fig. 8B, Table S6B). In ‘Hort16A’, β-ocimene and β-caryophyllene showed increased emission. Linalool and very high levels of DMNT were released after MeJA treatment in both cultivars. DMNT is derived from nerolidol by the action of a P450 enzyme (Lee et al. 2010).

The expression of TPS family members upon MeJA treatment was also investigated in leaves to see if there were any correlations with terpene accumulation and/or release (Fig. 8C). Transcript levels for eight AcTPS were up-regulated by MeJA treatment in one or both cultivars including transcripts for four sesquiterpene synthesis genes: *AcNES1, -2, AcAFS1* and *AcBCS,* that produce (*E*)-nerolidol, α-farnesene and β-caryophyllene respectively. α-farnesene was not detected at significantly higher levels in the treated samples, but the increased *AcNES1, -2* expression correlated well with the release of DMNT after MeJA treatmentwhile *AcNES3* was more lowly expressed. Increased β-caryophyllene also correlated with the increased *AcBCS* expression in ‘Hort16A’ tissue. For the monoterpene synthases, *AcLIS/NES* (linalool) expression was up-regulated in both cultivars upon MeJA treatment and correlated with the increased linalool accumulation and release, while *AcLS1* was only detected at low expression levels in ‘Hort16A’. In contrast *AcGES* expression was only significantly elevated in ‘Hort16A’ and correlated with increased geraniol accumulation in this cultivar.

### Terpene production and AcTPS expression in young fruit are also up-regulated by hormone treatment

To test the effect of defense-related phytohormones on terpene production and AcTPS gene expression in young fruit, 45 d old kiwifruit berries of ‘Red5’ were subjected to methyl jasmonate (MeJA), salicylic acid (SA) and a combined treatment (Figure S5). As shown in Figure S5, exogenous application of SA induced visible damage to the berry surface. With MeJA treatment no obvious visible phenotype was observed. When the berries were treated with the mixed solution of MeJA and SA, the SA-related damage to the surface of the fruit appeared to be largely alleviated. These results indicate that MeJA can effectively antagonize the damage induced by SA.

GC–MS results showed that MeJA-treated kiwifruit berries presented a significantly different terpene profile compared to buffer-treated controls. The terpene profiles of SA and SA + MeJA treated fruit were similar to the controls (Fig. 8D). The production of monoterpenes *α*-pinene, 1,8*-*cineole, linalool and geraniol were all significantly increased in the MeJA-treated berries. The sesquiterpenes *β*-caryophyllene and α-cubebene were also elevated. As in tissue-cultured leaves, (*E*)-nerolidol did not accumulate (< 1 ng∙g^−1^) in the tissue. These results indicate that MeJA treatment can activate terpene production in kiwifruit berries. In addition, transcript accumulation of multiple AcTPS genes were up-regulated by MeJA treatment, with six of them representing sesquiterpene synthesis genes: (*E*)-nerolidol (*AcNES1*, *AcNES2, AcNES3*, *AcNES/LIS*,), *β*-caryophyllene (*AcBCS*), *α*-farnesene (*AcAFS*) and germacrene synthase (*AcGDS*) (Fig. 8E). A subset of monoterpene biosynthesis related genes: *AcLS1*, *AcTPS1a*, *AcTPS1b*, were also induced after MeJA treatment (Fig. 8E). Of all the *AcTPS* genes tested, only *AcNES3* showed down-regulation in the MeJA-treated Red5 berries (Fig. 8E). Overall, exogenous application of MeJA on young fruit increased biosynthesis of a broad range of mono- and sesquiterpenes and in parallel induced expression of several *AcTPS* genes, while SA was able to antagonize terpene induction.

### Terpene production and AcTPS expression induced after herbivory by brown-headed leaf rollers

MeJA treatments of leaves and fruit indicated that terpenes and TPS gene transcripts could be induced via the JA-dependent signalling pathway. To establish if this observation could be extended to induction by economically important pests or pathogens of kiwifruit, experiments were undertaken to test the response of tissue-cultured leaves to herbivory by brown-headed leaf roller (BHLR, *Ctenopseustis obliquana*), a primary polyphagous pest of horticultural crops in New Zealand. Terpene accumulation inside the leaf tissue, as well as the emission/release of monoterpenes into the headspace, were both measured by GC–MS.

**Fig. 9:**
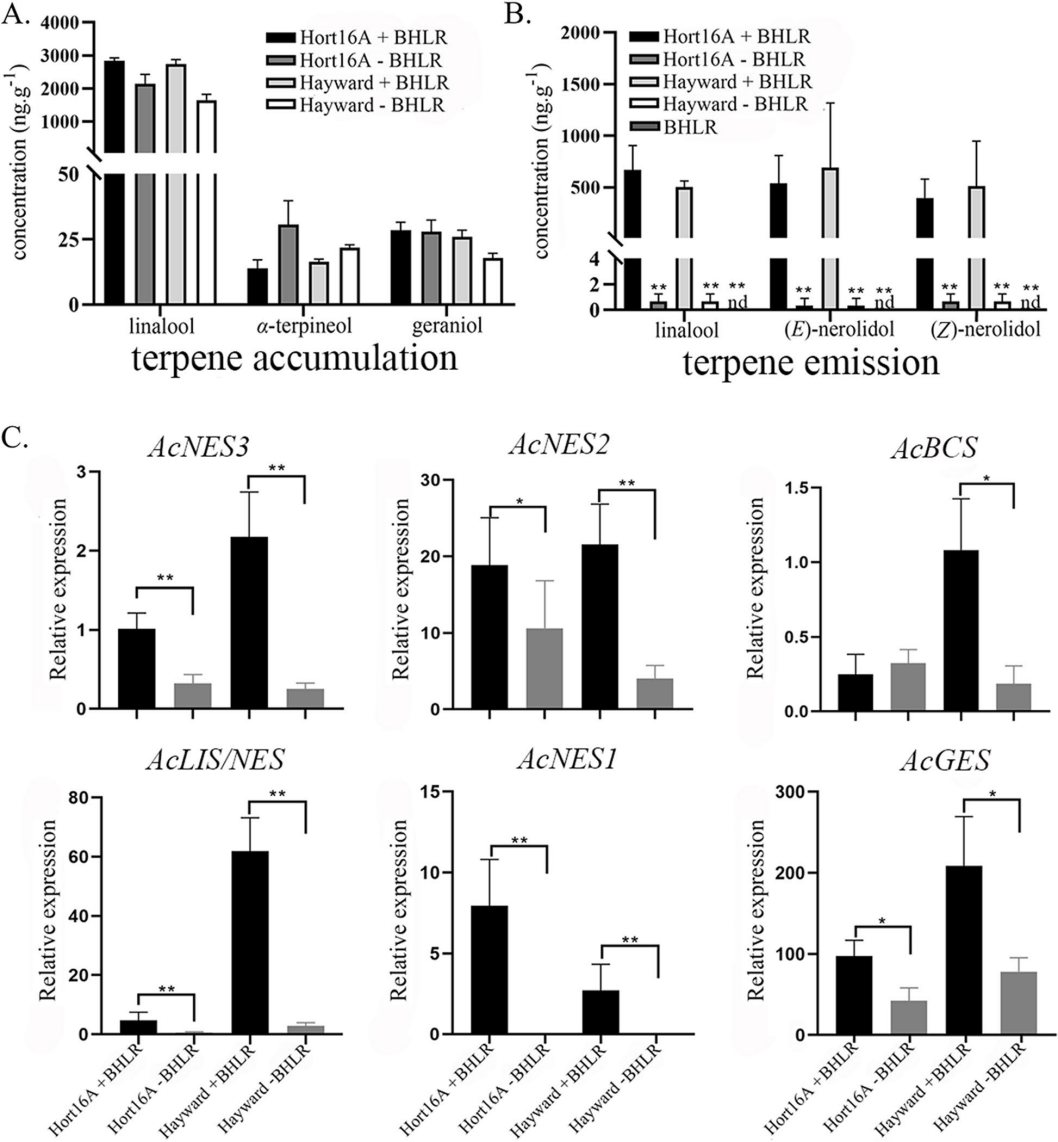
Terpenes produced and AcTPS gene expression changes after herbivory by brown-headed leaf rollers (BHLR). **A** Terpene accumulation in tissue culture leaves of kiwifruit ‘Hort16A’ and ‘Hayward’ either infested with BHLR or untreated control leaves. Leaf tissues were harvested after 6 d, ground to powder, and terpenes analysed by SPME GC–MS. **B** Terpene emission from tissue-cultured leaves of kiwifruit either infested with BHLR or untreated control. An insect-only sample (without plants, only insects, nutrients and agar) was also collected. Terpenes were collected by Tenax TA during 6 d feeding and analysed by GC–MS. **C** Transcript abundance of terpene synthase genes in infested and control leaves. Gene expression was determined by qRT-PCR. Data are means ± SE (*n* = 3). The Student’s t-test was used to test for statistical significance. Asterisks indicate a significant difference between caterpillar infested and untreated control leaves (*P < 0.05, **P < 0.01)

For both commercial cultivars tested (‘Hayward’ and ‘Hort16A’), linalool was again the predominant terpene accumulating in tissue. Slightly increased amounts of linalool accumulated in the herbivory samples of both cultivars versus controls (not significant at *P* < *0.05*) and > 50-fold smaller amounts of other monoterpenes such as α-terpineol and geraniol were present (Fig. 9A, Table S6C). When terpene emission was investigated, only very low levels of any terpenes were observed in healthy control plants (< 1 ng∙g^−1^ FW), and no terpenes were detected in the insect-only samples (Fig. 9B, Table S6D). However, upon insect herbivory, emission of linalool, (*E*)-nerolidol, and (*Z*)-nerolidol were significantly induced in insect-damaged plants compared to controls (Fig. 9B). DMNT was also consistently emitted from the herbivory samples (data not quantified) but at much lower levels (< 1 ng∙g^−1^ FW) compared to the MeJA treated samples and with no DMNT detected in control plants.

Similar to the MeJA treatments, herbivory significantly induced expression of some AcTPS genes (Fig. 9C). Consistent with increased (*E*)*-*nerolidol and (*Z*)*-*nerolidol release in both cultivars, *AcNES1*, *-2*, *-3* gene expression was also induced significantly in both cultivars upon herbivory. The increased linalool accumulation and emission upon herbivory correlated with increased TPS expression by the *AcLIS/NES* gene, whereas *AcGES* induction in both cultivars and *AcBCS* induction in ‘Hayward’ did not result in any significant increases in geraniol and/or β-caryophyllene production upon herbivory.

## Discussion

### The kiwifruit genome contains a small TPS family with members expressed in different organs and at different stages of development

A comparison of active TPS family members in kiwifruit with other horticultural crop plants such as tomato which contains 29 TPS (Zhou and Pichersky 2020), grape – 69 TPS (Martin et al. 2010) and apple – 10 TPS (Nieuwenhuizen et al. 2013) shows that the Red5 kiwifruit genome contains only a small-sized volatile TPS gene family. The present study identified 22 TPS gene models and a total of 15 predicted full-length and functional *AcTPS* genes, dominated by members of the TPS-b and -g subfamilies (Fig. 2). A single cluster of TPS-b gene models (*AcTPS16-20*, -*22*) assigned to chromosome 29 represents > 25% of TPS gene models identified in kiwifruit. Other TPS genes were present as homeologous pairs likely resulting from the ancient genome duplication/polyploidisation events in the *Actinidia* genome described by Wang et al. (2018).

The *Actinidia* genus has a reticulate polyploidy structure with diploids, tetraploids and hexaploids in diminishing frequency. All species have a haploid chromosome number of *n* = 29. The sequenced *A. chinensis* genomes from Red5 and ‘Hongyang’ and the sequenced *A. eriantha* genome from ‘Midao 31’ (Wang et al. 2023) are derived from diploid cultivars. Evidence for TPS tandem gene duplication and neofunctionalisation in the Red5 genome was seen at the *AcTPS1* locus on chromosome 29 where four closely related TPS genes produced different terpene products. Evidence for gene duplication and loss of function was suggested by the presence of TPS pseudogenes in the Red5 genome that appeared to be nonfunctional paralogues of neighbouring genes (Fig. 3). Orthologs of TPS genes found in other *Actinidia* species and cultivars were present in the Red5 genome and shared high sequence identity (> 90%). The terpene products produced by the five novel Red5 AcTPS genes described in this paper, the four previously characterised Red5 *AcTPS1a-d* genes, plus four TPS genes (*AaLS1*, *AcNES1*, *AdAFS1*, *AcGDS*) functionally characterised in other *Actinidia* species and cultivars can account for all the major volatile terpenes in Red5 fruit, flowers as well as vegetative tissues. Knowledge of Red5 TPS gene variation should allow identification of further allelic diversity within the *A. chinensis* gene space and functional homologs of Red5 TPS genes across the > 60 species in the *Actinidia* genus. However, the number of TPS genes and alleles present in kiwifruit cultivars with higher ploidy (e.g. hexaploid ‘Hayward’, tetraploid ‘Hortgem Tahi’) will be greater and likely more complex to study than in Red5, but their neofunctionalisation may serve as a source of novel genes allowing the plants to respond to different insects and pathogens.

Volatile analysis showed that terpene abundance and composition in kiwifruit is subject to spatial and temporal regulation, with big differences observed between different organs and tissues and during fruit development. This regulation is also reflected in the spatial and temporal gene expression patterns observed for each of the AcTPS-R5 members present in the genome. For example, high levels of gene expression were observed for five AcTPS-R5 genes in the peel and flesh of unripe fruit, often peaking at 75–90 days post anthesis. Other family members showed quite specific expression in leaves (*AcTPS1d*), roots (*AcTPS15*) or buds (*AcTPS1c*). The in planta function of terpene synthases is driven by their access to GDP from the MEP pathway in the plastids versus FDP derived from the mevalonate pathway in the cytosol/ER/peroxisome. Subcellular location of the AcTPS enzymes was used to verify which substrate pool was available to each TPS enzyme (Fig. 7). This analysis was particularly helpful in clarifying the function of *AcLIS/NES* as a linalool synthase in planta, despite the enzyme producing nerolidol more efficiently in kinetic studies.

### Biological roles and functional redundancy within the TPS family in kiwifruit

**Fig. 10:**
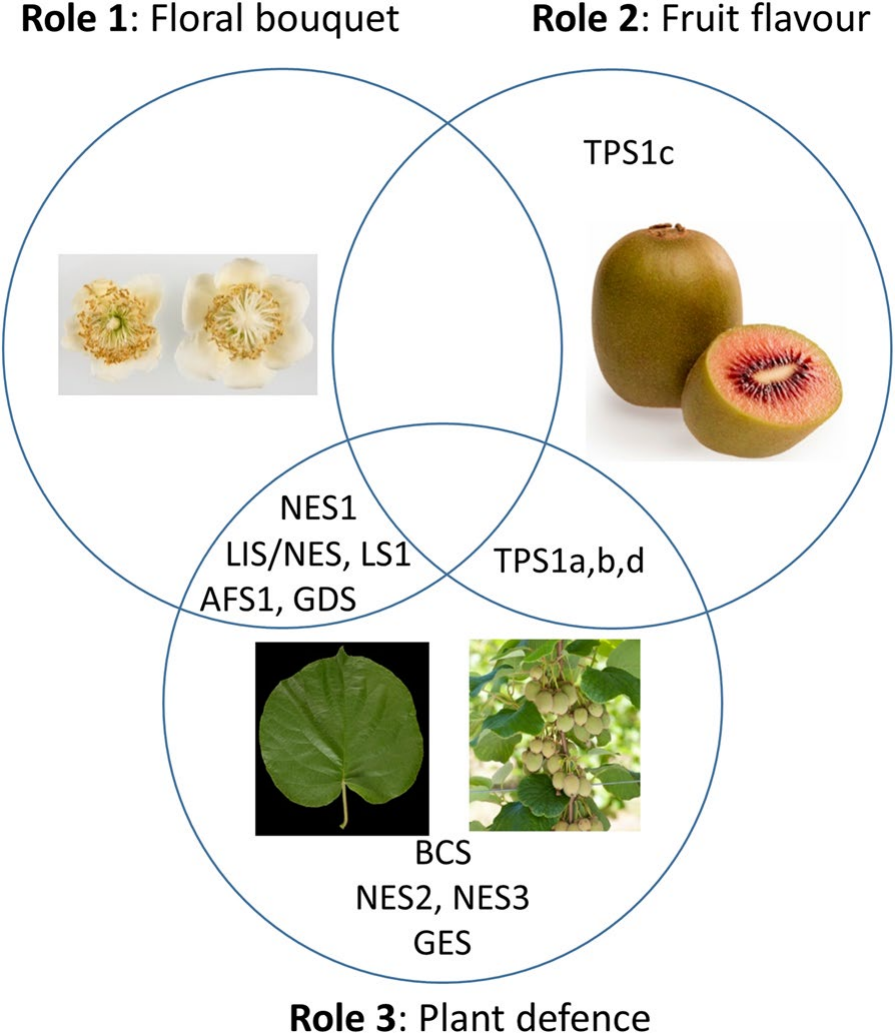
Overlapping functions of *Actinidia* terpene synthases. TPS genes were classified based on their expression profiles and likely functional roles in flowers, ripe fruit, leaves and young fruit. Role 1: TPS producing terpenes that form part of the floral bouquet that helps to attract pollinators (five genes). Role 2: TPS producing terpenes that contribute to fruit flavour and aroma to attract frugivores and facilitate seed dispersal (four genes). Role 3: TPS induced by MeJA or herbivory and generating terpenes involved in plant defence against pests and diseases (twelve genes). Intersections indicate the overlapping functions

One role of terpenes in kiwifruit biology can be found in the flowers, where they form part of the floral bouquet that helps to attract pollinators (Fig. 10). Several TPS have been identified previously in *Actinidia* that are highly expressed in floral organs and produce specific blends of terpenes. For example *AdAFS1* and *AdGDS1* in ‘Hayward’ produce α-farnesene and germacrene D respectively (Nieuwenhuizen et al. 2009) whilst *AcNES1* and *AaLS1* produce (*E*)-nerolidol and linalool respectively in ‘Hort16A’ and ‘Hortgem Tahi’ (Chen et al. 2010; Green et al. 2012). In Red5, (*E*)-nerolidol and linalool are abundant volatiles produced in flowers, but interestingly the TPS gene correlated with linalool production in Red5 flowers (*AcLIS/NES*) is different to *LS1* that produces floral linalool in at least two other *Actinidia* species – *A. arguta* and *A. polygama* (Chen et al. 2010).

A second role of terpenes in kiwifruit biology is the attraction of frugivores (seed dispersal animals) by contributing to the taste and aroma blend of ripe fruit (Fig. 10). The *TPS1* locus on chromosome 29 seems to fulfil a particularly dominant role in this function with several genes and alleles producing different terpenes in ripe fruit having now been characterised from multiple species (Davidson et al. 2023; Nieuwenhuizen et al. 2015; Wang et al. 2021; Zeng et al. 2020). The diversity of compounds produced from this single locus may serve as a source of novelty to breed kiwifruit with new aromas and flavours.

The third role for terpenes in kiwifruit examined in this paper is in plant defence (Fig. 10). From the MeJA treatment experiments it is apparent that jasmonate signalling forms an important part of the defence arsenal in kiwifruit. Jasmonate is known to be very rapidly produced upon herbivory (Kessler and Baldwin 2002) and we show that a subset of terpene synthase genes is induced by MeJA treatment of leaves and young fruit (Fig. 8). Interestingly DMNT emission was much higher in MeJA treated tissue-cultured plants, while its (*E*)-nerolidol precursor was much lower, compared to BHLR infested plants where the inverse was observed. This observation points to other factors being important too in the biotic interactions, such as actual physical leaf damage and insect saliva factors that may suppress or modify plant defences.

When considering all the TPS expression profiles and functional data together, it becomes apparent that roles in pollinator attraction, seed dispersal and plant defence are not mutually exclusive and a subset of terpene synthases likely fulfil overlapping functions (Fig. 10). For example, nerolidol synthase (*NES1*) and α-farnesene synthase (*AFS1*), first identified as important contributors to the floral aroma in ‘Hort16A’ and ‘Hayward’, respectively are also highly expressed in Red5 cane and young fruit (Fig. 4), as well as being induced by MeJA and/or BHLR infestation. Similarly, while the *TPS1* genes are part of the complex ripe fruit aroma locus (Zeng et al. 2020), *AcTPSd* also shows high expression in leaves (Fig. 4). Several examples of functional redundancy/convergence were also observed. Three nerolidol synthases that are part of distinct TPS subfamily lineages were all induced by herbivory (*NES1-3*), while two linalool synthases from different TPS subfamilies were associated with floral roles (*LS1*, *LIS/NES*). The comprehensive TPS gene identification, expression and functional analysis presented in this paper allows the wider picture of overlapping roles and functional redundancy to be observed in *Actinidia* for the first time.

### Accumulation vs emission of terpenes in kiwifruit defence

Apart from basal levels of defence, induced defences are an important mechanism for plants to minimise the cost of defence, while maximising the return. In kiwifruit, until now, little was known about defence-related terpenes and their synthases. In vegetative tissues, we observed several terpenes and their synthases to be induced upon herbivory by the economically relevant pest BHLR. This included linalool, (*E*)- and (*Z*)-nerolidol and derivatives such as linalool oxides, and the homoterpene DMNT and the concomitant induction of AcTPS genes such as *AcLIS/NES*, and several *AcNES* genes*. AcNES1* has previously been shown to produce both nerolidol isomers in vitro but ‘Hort16A’ flower extracts contained only the (*E*)-isomer (Green et al. 2012).

By comparing ‘in tissue’ accumulation versus headspace emission, unique distributions of volatiles were uncovered. For example linalool was identified to both accumulate and to be released into the headspace, while (*E*)- and (*Z*)-nerolidol and DMNT were exclusively found in the headspace. This indicates that these latter compounds are likely produced mainly de novo upon herbivory and only at the site of feeding by up-regulation of TPS expression. Linalool on the other hand is already present at significant levels pre-herbivory and appears to be further induced by up-regulation of linalool TPS expression.

Fleshy fruit have evolved from their dry fruit ancestors and the fleshy parts serve functions in protecting, nurturing and dispersing of seeds. Fleshy fruit types are thought to have co-evolved with endozoochory (seed dispersal through ingestion by animals) and are often associated with shaded habitats (Lorts et al. 2008). The aroma of ripe kiwifruit may have evolved as an adaptation to attract mammals such as bears, monkeys and martens to consume the fruits and consequently disperse their seeds (Naoe et al. 2019; Nevo et al. 2015). These dispersal functions of terpenes were preceded by pollination attraction in most flowering insect-pollinated plants and by defence against pest and diseases in all plants. Identifying the full set of Red5 TPS genes and their overlapping functions provides a framework to better understand the volatile terpene language within the *Actinidia* genus and in other horticultural crops and how the multiple functions of terpene synthases have evolved to support the various biological and ecological roles. Armed with this knowledge we may be able to target specific terpenes in breeding to simultaneously improve kiwifruit flavour/aroma and resistance to pests.

## Methods

### Plant and insect material

Red5 fruit for analysis through development were obtained from Plant & Food Research (PFR) orchards in Motueka, New Zealand. A detailed description of these samples including the size, weight, dry matter content, and a representative photograph is given in Zeng et al. (2020). Additional trays of young Red5 fruit were harvested at 45 d post anthesis from PFR orchards in Motueka for hormone treatment. Other Red5 tissues were collected from plants grown under ambient conditions in PFR glasshouses in Auckland, New Zealand. Tissue-cultured plants ~ 3–5 cm tall of ‘Hort16A’ and ‘Hayward’ were provided by Multiflora Laboratories (Auckland, New Zealand). *N. benthamiana* plants were grown in a PFR glasshouse at 25 °C with a 16 h light/8 h dark photoperiod. Brown-headed leaf rollers (BHLR, 3^rd/^4^th^ instar) (*Ctenopseustis obliquana*) were raised by as described in Barrington et al. 1993.

### Tissue and dynamic headspace volatile analysis by GC–MS

Frozen kiwifruit tissues from developing fruit (2–3 g), flower (3–4 g), leaves (1–2 g), bud (1–2 g) and tobacco leaves (1–2 g) were ground in triplicate with liquid nitrogen and 30% (w/w) NaCl was added. Volatile analysis of the tissues by SPME GC–MS was conducted according to Zeng et al. (2020) with the minor modifications. Volatiles were collected at 40 °C for 10 min with agitation, controlled by a multipurpose sampler injection system (Gerstel Mülheim, Germany). SPME fibres (1 cm) coated with 50/30 μm DVB/CAR/PDMS were used for the volatile collection. Separation was effected using a 30 m × 0.25 mm internal diameter × 0.25 μm film thickness DB-WAX UI (Agilent, Santa Clara, CA) capillary GC column in an Agilent7890 GC coupled to a Leco BT time-of-flight mass spectrometer (Leco Corp., St Josephs, MI). Terpenes were semi-quantified using the single *m/z* 93 ion and identified by comparison with the National Institute of Standards and Technology (NIST) database (version V2.3, 2017; in-house mass spectral libraries and confirmed by comparison of retention indices with those of authentic standards and literature values. The sample peak areas were converted into ng∙g^−1^ fresh weight (FW) by comparison with the internal standard cyclohexanone (2.025 μg per sample) or hexadecane standard added in each sample. All the treatments consisted of at least three replicates.

For dynamic headspace analysis, the emitted volatiles were trapped onto Tenax TA matrix (100 mg of Tenax TA 80–100 mesh, Sigma-Aldrich, St. Louis, MO) (See M&M sections ‘MeJA treatment’ and ‘Herbivore treatment’ respectively for details) and samples were eluted with 1 mL of pentane/ether(1:1 v/v). Afterwards, 50 µg of cyclohexanone was added as the internal standard and the eluted volatile solvent extract was concentrated tenfold under a stream of N_2_ gas. Extracts (1 µL) were loaded onto the GC–MS and analysed as described in Zeng et al. 2020. Terpene peaks were semi-quantified using single *m/z* 93 ion according to the method described in the above paragraph.

### Identification and phylogenetic analysis of kiwifruit TPS genes

TPS sequences were identified using BLASTP against the manually annotated *A. chinensis* var. *chinensis* Red5 genome available from ftp://ftp.ncbi.nlm.nih.gov/genomes/all/GCA/ 003/024/255/GCA_003024255.1_ Red5_PS1_1.69.0. Sequences were manually curated, aligned using ClustalW, and analysed for known conserved motifs and features using the Geneious software (v. R10). Phylogenetic trees were constructed using MEGA program v7.0.14.

### RNA isolation, cDNA synthesis and quantitative real-time PCR

Total RNA was isolated using the Spectrum plant total RNA extraction Kit (Sigma-Aldrich, St. Louis, MO). The RNA was qualified by NanoDrop 1000 UV spectrometry and the quality verified by agarose gel electrophoresis. 10 U DNase I (Roche Applied Science, Mannheim, Germany) was applied to each sample to remove the genomic DNA, and for each sample cDNA was made from 1 µg total RNA using a QuantiTect Reverse Transcription kit (QIAGEN, Hilden, Germany), according to the manufacturer’s protocol. The cDNA used for qRT-PCR was diluted 50-fold prior to amplification. qRT-PCR gene expression analysis for TPS genes was performed alongside the combined reference genes *EF1α* (Nieuwenhuizen et al. 2009) and *Actin* (Walton et al. 2009) on a LightCycler 480 platform using the LightCycler 480 SYBR Green master mix (Roche Applied Science, Mannheim, Germany) in a reaction volume of 5 µL. Results were analysed using the LightCycler 480 software (Roche Applied Science, Mannheim, Germany). The amplification program was: 5 min at 96 °C; 40 cycles of 10 s at 95 °C, 10 s at 60 °C, and 20 s at 72 °C; followed by melting curve analysis: 95 °C 5 s, 65 °C 60 s, then ramping at 0.18 °C∙s^−1^ to 95 °C. The results were analysed using the ΔΔCt method with primer amplification efficiency corrections and using the geometrical mean of the two reference genes (Vandesompele et al. 2002). Primer sequences and efficiencies (determined by the serial dilution method) are listed in Table S7. Three biological and four technical replicates were conducted in each real-time PCR analysis.

### Cloning of full-length TPS genes

Full-length clones of *AcBCS*, *AcNES2* and *AcNES3* were amplified from Red5 cDNA (75 + 150 days peel sample) using primers to the full-length ORFs predicted by the gene models for these genes given in Table 1. The 3’ primer for *AcGES* was obtained at the C-terminus of the gene model Acc26061. The 5’ primer was designed after extending the predicted ORF by 105 aa at the N-terminus using publicly available RNAseq data (Figure S1, http://kiwifruitgenome.org/jbrowse/). N- and C-terminal primers for *AcLIS/NES* primers were designed to the truncated gene models of Acc19057 and Acc19058 combined (Figure S1). For *AcGES* and *AcLIS/NES,* cDNA from Red5 peel (60 d) were used as template*.*

### Transient overexpression of kiwifruit TPS genes in *N. benthamiana* leaves

Full-length AcTPS ORFs were recombined into the Gateway sequencing vector pDONR221 (Invitrogen) using the primers listed in Table S7. After full-length sequence confirmation, genes were cloned into the pHEX2 binary vector (Hellens et al. 2005) by Gateway LR reactions to generate the over-expression constructs pHEX2_AcTPS (CaMV 35S:ORF:ocs-3’). Transient overexpression of AcTPS genes in *N. benthamiana* leaves was performed as previously described (Zeng et al. 2020), but using a final concentration of bacteria of OD600 = 0.5. Leaves infiltrated with a pHEX2_GUS construct were used as negative controls (Nieuwenhuizen et al. 2009). pHEX2_AcDXS (Nieuwenhuizen et al. 2015) or pEAQ-tHMGR-2A-BCCP1 (Lee et al. 2019) were co-infiltrated with each TPS construct and pHEX2_GUS to increase substrate production for synthesis of terpenoids. *N. benthamiana* leaves (1–2 g) were harvested at 7 d post infiltration, ground in liquid nitrogen, and stored at -80 °C. Terpene volatiles were collected by SPME and analysed by GC–MS. All the experiments were conducted with at least three to six biological replicates.

### Expression of AcTPS in *Escherichia coli*

The complete ORF of *AcNES2, AcNES3, AcBCS* and *AcGES,* including any potential ChloroP-predicted plastid targeting signal peptides (Emanuelsson et al. 2000), were amplified by PCR and cloned into the pET300 vector (N-terminal HIS-tag, Invitrogen). *AcLIS/NES* was cloned into the pMAL-c6T vector (NEB) to express Mal-BP-TPS recombinant protein. Primers used for PCR amplification are shown in Table S7. Protein expression and purification was done as described previously (Zeng et al. 2020). The purity of the recombinant proteins was confirmed by SDS-PAGE (Figure S3) and protein concentration was calculated by NanoDrop 2000. Samples (2.5 mL volume) were desalted using a PD10 column (GE-Pharmacia) eluted in 3.5 mL buffer (containing 20 mM HEPES, 150 mM NaCl, 1 mM DTT, pH 7.5). Samples were stored in 10% (v/v) glycerol in small aliquots at -80 °C until further use.

### Analysis of terpene production by recombinant AcTPS

TPS enzyme assays were performed as described previously (Zeng et al. 2020). In short, purified recombinant enzyme (2–50 μg protein) was added to 2 mL of an optimised TPS assay buffer (50 mM Bis–Tris Propane pH 7.5, 10 mM MgCl_2_, 10% glycerol (v/v) and 5 mM DTT). Samples were incubated (30 °C for 1 h, whilst shaking) using 50 μM GDP or FDP as substrates.

Kinetic parameters *Vmax*, *Km* and *Kcat* of each AcTPS were calculated in the presence of GDP and FDP by non-linear least square fitting of the data to the Michaelis–Menten equation using the GraphPad prism 8.0 software (San Diego, CA, U.S.A). Kinetic parameters for each TPS were calculated using the major product peak. Reactions were initiated by adding 1 µg of affinity-purified HIS-tagged or MAL-MBP-tagged enzyme in a final volume of 1 mL of optimised assay buffer (pH 7.5) and varying concentrations (0.5 μM-50 μM) of substrates (FDP or GDP). The assays were incubated at 30 °C for 1 h, then samples were snap frozen at -80 °C until analysis. For both TPS enzyme assays and kinetic analysis, headspace terpenes were collected by SPME and analysed by GC–MS. Hexadecane was used as the internal standard.

### Subcellular localisation of AcTPS genes

Complete AcTPS ORFs were amplified without a stop codon (using primers in Table S7) and inserted into the pMDC83-GFP vector (Curtis and Grossniklaus 2003) by Gateway cloning. Translational fusions were infiltrated into *N. benthamiana* leaves and protoplasts produced as described in Zeng et al. (2020). Fluorescence images were acquired on a confocal laser-scanning microscope (TCS SP8; Leica, Wetzlar, Germany) using previously described excitation wavelengths and detection windows (Zeng et al. 2020). All fluorescence experiments were repeated independently at least three times. AcTPS-GFP signals were merged with chlorophyll autofluorescence signals or red fluorescence signals from pMDC43-RFP to verify chloroplastic and cytoplasmic subcellular localisation respectively.

### Hormone treatments and volatile sampling of tissue-cultured leaves and young fruit

Tissue-cultured kiwifruit plants ‘Hort16A’/’Hayward’ were grown in ½ MS medium + 0.8% agar at 21 °C with a 14 h photoperiod. When 4–8 cm tall, the plants were transferred to 1 L headspace sampling jars containing ½ MS + agar 0.8% (w/v). Leaves were lightly sprayed with 200 µM solution of MeJA or control solution (0.125% Triton X) and allowed to air dry. Dried air was provided to the jar at a constant flow rate of 30 mL∙min^−1^ for 6 d and the exit air was sampled continuously using a Tenax TA 80/100 mesh (100 mg) cartridge. At completion, terpenes were eluted with 1 mL pentane:ether (1:1) and analysed by GC–MS as described above in the M&M section ‘Tissue and headspace volatile analysis by GC–MS’. At completion of the sampling, leaf tissues were also collected for SPME GC–MS tissue analysis as described above and for qRT-PCR gene expression analysis.

Twenty young Red5 fruit (45 d after flowering) were either dipped for 10 min in 200 µM MeJA solution (including 0.125% Triton X-100), 10 mM salicylic acid or a combination of both. Control fruit were dipped into Triton solution (0.125% Triton X-100 only) and fruit were allowed to air dry. Twenty-four hours post-treatment, fruit were photographed and peel tissue of treated and control samples was snap frozen immediately in liquid nitrogen and stored at − 80 °C for further qRT PCR gene expression and tissue volatile accumulation analysis (SPME GC–MS).

### Herbivore treatment and volatile sampling

Four tissue-cultured kiwifruit plants ‘Hort16A’/’Hayward’ (4–8 cm as described in ‘MeJA treatment’) per 1 L pot were infested with 10 BHLR (3^rd^ or 4^th^ instar). Dried air was provided to the headspace sampling jars as describe for MeJA treatment and samples collected on Tenax TA (100 mg) cartridges for 6 d. Terpenes were eluted with 1 mL pentane:ether (1:1) and analysed by GC–MS as described above. At completion of the sampling, tissues were also collected for SPME GC–MS analysis and qRT-PCR as described above. Details of the experimental set-up are shown in Figure S4.

### Supplementary Information

**Additional file 1: Table S1.** Complete dataset of terpene volatiles in Red5 tissues. **Table S2A.** Features of the 22 AcTPS gene models identified in the Red5 genome. **Table S2B.** Published AcTPS genes and corresponding Red5 gene models. **Table S3.** Abbreviation, full gene name and GenBank accession number for TPS used in this study. **Table S4.** Headspace volatile terpenes produced by transient expression of AcTPS genes in planta*. ***Table S5.** Volatile terpenes produced by heterologous expression of AcTPS genes in *E. coli. ***Table S6.** Terpenes produced by *Actinidia *leaves after MeJA treatment (S6A, B) or herbivory by brown-headed leaf roller (S6C, D). **Table S7. **Primers used in this study.

**Additional file 2: Figure S1. **Cloning strategy for *AcGES* and *AcLIS/NES* based on RNAseq coverage and gene models. **Figure S2.** Amino acid alignment of full length AcTPS genes identified in the Red5 genome. **Figure S3.** SDS-PAGE analysis of purified recombinant His-tagged and Mal-tagged AcTPS proteins. **Figure S4.** Experimental setup for herbivore treatment of kiwifruit leaves. **Figure S5.** Hormone treatment of young Red5 fruit.

## Data Availability

The authors confirm that the data supporting the findings of this study are available within the article and/or its supplementary materials.

## Abbreviations

AA: Amino acid
BHLR: Brown-headed leaf roller (*Ctenopseustis **obliquana*)
CYP: Cytochrome P450 monooxygenase
DMAPP: Dimethylallyl diphosphate
DMNT: (*E*)-4,8-Dimethyl-1,3,7-nonatriene
FDP: Farnesyl diphosphate
GDP: Geranyl diphosphate
HIPVs: Herbivore induced plant volatiles
IDP: Isopentenyl diphosphate
MeJA: Methyl jasmonate
MEP: Methylerythritol phosphate
MVA: Mevalonic acid
ORF: Open reading frame
qRT-PCR: Quantitative reverse transcription PCR
SPME: Solid phase micro extraction
TMTT: 4,8,12-Trimethyltrideca-1,3,7,11-tetraene
TPS: Terpene synthase
