# Supplementary material for: The terpene synthase (TPS) gene family in kiwifruit shows high functional redundancy and a subset of TPS likely fulfil overlapping functions in fruit flavour, floral bouquet and defence
Source: Mol Hortic. 2023 May 8;3:9. doi: 10.1186/s43897-023-00057-0 (PMC10514967; doi:10.1186/s43897-023-00057-0)
Supplement: Supplementary file 1 — Additional file 1: Table S1. Complete dataset of terpene volatiles in Red5 tissues. Table S2A. Features of the 22 AcTPS gene models identified in the Red5 genome. Table S2B. Published AcTPS genes and corresponding Red5 gene models. Table S3. Abbreviation, full gene name and GenBank accession number for TPS used in this study. Table S4. Headspace volatile terpenes produced by transient expression of AcTPS genes in planta. Table S5. Volatile terpenes produced by heterologous expression of AcTPS genes in E. coli. Table S6. Terpenes produced by Actinidia leaves after MeJA treatment (S6A, B) or herbivory by brown-headed leaf roller (S6C, D). Table S7. Primers used in this study. [file 43897_2023_57_MOESM1_ESM.docx]

|  | 1, 8-cineole | linalool | 4-terpineol | bornylene | *p*-cymeme | α-terpinene | terpinene-4-ol | limonene | myrcene |
| --- | --- | --- | --- | --- | --- | --- | --- | --- | --- |
| leaf | / | 1951.1±117.7 | / | / | / | / | / | / | / |
| bud | / | 1531.7±170.7 | 12.1±0.7 | / | / | 16.6±1.0 | 22.7±2.2 | 122.4±7.7 | / |
| flower | / | 2800.4±419.9 | 0.4±0.4 | / | / | / | / | / | / |
| 45 d | 35.4±13.9 | 10.8±5.1 | 0.4±0.4 | 6.8±3.1 | 0.3±0.1 | / | 0.6±0.2 | / | / |
| 60 d | 25.7±4.8 | 26.3±6.1 | 2.2±0.6 | 11.9±2.9 | 0.2±0.0 | / | / | / | / |
| 75 d | 86.1±18.3 | 28.0±3.8 | 4.4±0.7 | 18.5±3.1 | 0.1±0.1 | / | 0.4±0.4 | / | / |
| 90 d | 129.8±29.6 | 28.8±2.5 | 5.2±0.8 | 15.0±1.3 | 0.2±0.2 | / | 1.9±0.3 | / | 0.6±0.6 |
| 120 d | 120.0±46.7 | 1.9±0.4 | 1.3±0.3 | 1.1±0.4 | 1.6±0.6 | 0.7±0.1 | 1.3±0.5 | 1.4±0.6 | 0.1±0.1 |
|  |  |  |  |  |  |  |  |  |  |
|  | sabinene | α-pinene | β-pinene | β-caryophyllene | (*E*)-nerolidol | farnesol | citronellal | geraniol |  |
| leaf | / | / | / | / | / | / | / | / |  |
| bud | / | 5.1±1.6 | 5.2±1.9 | 14.7±1.8 | / | / | / | 848.0±93.6 |  |
| flower | / | / | / | / | 122.9±1.8 | 470.5±88.7 | 7.7±4.1 | / |  |
| 45 d | / | / | 1.0±0.3 | 6.8±2.7 | 0.2±0.0 | / | / | / |  |
| 60 d | / | / | 1.2±0.1 | 3.4±0.3 | / | / | / | / |  |
| 75 d | / | / | 1.9±0.5 | 5.8±1.9 | / | / | / | / |  |
| 90 d | / | / | 1.9±0.4 | / | / | / | / | / |  |
| 120 d | 1.9±0.7 | 2.2±0.5 | 3.5±0.7 | / | / | / | / | / |  |

**Table S1.** Complete dataset of terpene volatiles in Red5 tissues.

Terpene volatiles were collected by SPME and analysed by GC-MS from developing fruit peel at 45–120 days (d) after anthesis. Leaves were sampled at the mature, full-expanded stage. Buds were harvest at less than 2 cm. Flowers were collected when fully open and included petals, stamens and ovaries. Terpene concentrations are in ng∙g^-1^. Data are means ± SE (n = 3).

| Red5 Gene model | Classification | TPS clade | Exons | Chrom | Protein length | Start | Stop | Strand | RRX8W | DDXXD | NSE/DTE | BLASTp | Comment |
| --- | --- | --- | --- | --- | --- | --- | --- | --- | --- | --- | --- | --- | --- |
| Acc09580 | AcTPS1-R5 | e/f | 12 | 8 | 776 | 22530572 | 22535143 | F | no | yes | yes | (*E,E*)-geranyl linalool synthase | Full length |
| Acc09993 | AcTPS2-R5 | a | 9 | 9 | 521 | 2654903 | 2671249 | F | no | yes | yes | (-)-germacrene D synthase | missing exon 1 (50 aa N-term) |
| Acc13525 | AcTPS3-R5 | g | 7 | 12 | 546 | 10828965 | 10831896 | R | yes | yes | yes | terpene synthase | Full length |
| Acc13740 | AcTPS4-R5 | a | 8 | 12 | 483 | 15647527 | 15653993 | F | no | yes | yes | (-)-germacrene D synthase | Not full length, exons missing |
| Acc13742 | AcTPS5-R5 | a | 7 | 12 | 561 | 15709563 | 15715335 | F | yes | yes | yes | (-)-germacrene D synthase | Full length |
| Acc15004 | AcTPS6-R5 | g | 5 | 13 | 400 | 15273785 | 15277021 | F | no | yes | yes | terpene synthase | Not full length |
| Acc15182 | AcTPS7-R5 | c | 9 | 13 | 824 | 17948187 | 17960778 | R | no | yes | no | ent-copalyl diphosphate synthase | Full length |
| Acc17359 | AcTPS8-R5 | e/f | 12 | 15 | 768 | 13865688 | 13870452 | F | no | yes | no | (*E,E*)-geranyl linalool synthase | Full length |
| Acc19057 | AcTPS9-R5 | g | 2 | 17 | 158 | 3122266 | 3123441 | R | yes | no | no | terpene synthase | Not full length |
| Acc19058 | AcTPS10-R5 | g | 3 | 17 | 199 | 3127947 | 3130412 | R | no | yes | yes | terpene synthase | Not full length |
| Acc19469 | AcTPS11-R5 | a | 7 | 17 | 565 | 14283940 | 14279400 | R | no | yes | yes | germacrene D synthase | Not full length |
| Acc20590 | AcTPS12-R5 | g | 7 | 18 | 285 | 15968351 | 15971152 | F | no | no | no | (*3S,6E*)-nerolidol synthase | Not full length |
| Acc20592 | AcTPS13-R5 | g | 7 | 18 | 590 | 15995705 | 15998499 | F | no | yes | yes | (*3S,6E*)-nerolidol synthase | Full length |
| Acc25053 | AcTPS14-R5 | g | 7 | 22 | 573 | 14197699 | 14200433 | F | no | yes | yes | (*3S,6E*)-nerolidol synthase | Full length |
| Acc26061 | AcTPS15-R5 | g | 8 | 23 | 470 | 8530994 | 8535397 | F | no | yes | yes | terpene synthase | Not full length |
| Acc32631 | AcTPS16-R5 | b | 7 | 29 | 609 | 1189455 | 1194116 | F | yes | yes | yes | (-)-alpha-terpineol synthase | Full length |
| Acc32632 | AcTPS17-R5 | b | 7 | 29 | 441 | 1198042 | 1205423 | F | yes | yes | yes | (-)-alpha-terpineol synthase | Not full length |
| Acc32633 | AcTPS18-R5 | b | 3 | 29 | 282 | 1221425 | 1223547 | F | yes | yes | no | myrcene synthase | Not full length |
| Acc32635 | AcTPS19-R5 | b | 7 | 29 | 559 | 1252569 | 1257711 | F | yes | yes | yes | (-)-alpha-terpineol synthase | Not full length |
| Acc32636 | AcTPS20-R5 | b | 7 | 29 | 608 | 1279500 | 1286365 | F | yes | yes | yes | (-)-alpha-terpineol synthase | Full length |
| Acc33493 | AcTPS21-R5 | e/f | 14 | 24 | 793 | 2121094 | 2128487 | F | no | yes | yes | ent-kaur-16-ene synthase | Full length |
| Acc33737 | AcTPS22-R5 | unknown | 2 | 30* | 159 | 1193355 | 1286057 | R | no | no | yes | (-)-alpha-terpineol synthase | Not full length |

**Table S2A.** Features of the twenty-two AcTPS gene models identified in the Red5 genome.

**Red5 Gene model**: The official IASMA (Instituto Agrario San Michele all'Adige) ‘gene model 1’ ID in the Red5 kiwifruit genome (http://plants.ensembl.org/Actinidia_chinensis/Info/Index). **Classification**: AcTPS-R5 nomenclature for the twenty-two terpene synthase (TPS) gene models identified in the Red5 genome. **TPS clade**: classification of the TPS gene models (TPS-a to TPS-h) according to phylogenetic and functional studies (Chen *et al*., 2011). **Exons**: number of annotated exons for TPS gene model (IASMA gene model 1). **Chrom**: Chromosomal location in the Red5 genome. n/a, not assigned to a chromosome. **Protein length**: Predicted amino acid number of the TPS open reading frame. **Start**: First position of the TPS coding sequence in the chromosome. **Stop**: Last position of the TPS coding sequence in the chromosome. **Strand**: ‘F’ forward (+) strand and ‘R’ reverse (-) strand relative to the chromosome annotation. **RRX8W**: ‘Yes’ means exact motif is present in the predicted position (end of exon 1) of TPS gene model. ‘No’ means that the corresponding part of the gene is absent (probable pseudogene) or that the motif is absent from this class. **DDXX(D/E)**: ‘Yes’ means the divalent metal ion (typically Mg2+) binding motif is present either as ‘DDXXD’ or ‘DDXXE’ at the expected position at the end of exon 4. ‘No’ means that the corresponding part of the gene or the motif is absent (probable pseudogene). Variations of the ‘DDXX(D/E) are shown as is the equivalent metal binding region in the predicted TPS-c gene models which do not possess the aspartate rich motif. The NSE/DTE metal binding motif is based on the consensus: (L,V)(V,L,A)(N,D)D(L,I,V)X(S,T,G)XXXE (Christianson, 2006; Zhou and Peters, 2009). **BLASTp**: Best hit in the GenBank non-redundant (NR) protein database. **Comment**: Yellow = Gene models are incomplete and likely represent pseudogenes. Green = Gene models that are incorrectly annotated and should be joined together. Grey = Gene model is incomplete, but N-terminus is present in the genome but not annotated. Blue = Gene models at a complex *AcTPS1* locus on chromosome 29 that have not been correctly resolved (Zeng *et al*., 2020). * = part of an unanchored contig that may be part of the complex locus on chromosome 29.

| Published Name | GenBank accession number | Main product | Reference | Red5 Gene model | TPS clade | Exons | Chrom | Protein length | RRX8W | DDXXD | NSE/DTE |
| --- | --- | --- | --- | --- | --- | --- | --- | --- | --- | --- | --- |
| AcTPS1a | MK809270 | sabinene | Zeng et al., 2020 | Acc32633 | b | 7 | 29 | 603 | yes | yes | yes |
| AcTPS1b | MK809271 | 1,8-cineole | Zeng et al., 2020 | Acc32633 | b | 7 | 29 | 603 | yes | yes | yes |
| AcTPS1c | MK809272 | geraniol | Zeng et al., 2020 | Acc32633 | b | 7 | 29 | 603 | yes | yes | yes |
| AcTPS1d | MK809273 | diterpene | Zeng et al., 2020 | Acc32635 | b | 7 | 29 | 605 | yes | yes | yes |
| AdGDS | AY789791 | germacrene D | Nieuwenhuizen et al., 2009 | Acc19469 | a | 7 | 17 | 565 | yes | yes | yes |
| AdAFS1 | FJ265785 | α-farnesene | Nieuwenhuizen et al., 2009 | Acc17359 | e/f | 12 | 15 | 768 | no | yes | yes |
| AaLS1 | GQ338153 | linalool | Chen et al. 2010 | Acc20592 | g | 7 | 18 | 574 | no | yes | yes |
| AaTPS1 | KF319036 | α-terpinolene | Nieuwenhuizen et al., 2015 | Acc32635 | b | 7 | 29 | 604 | yes | yes | yes |
| ApLS1 | GQ338154 | linalool | Chen et al. 2010 | Acc20592 | g | 7 | 18 | 574 | no | yes | yes |
| AcTPS1 | KF319035 | β-mycrene | Nieuwenhuizen et al., 2015 | Acc32633 | b | 7 | 29 | 603 | yes | yes | yes |
| AcNES1 | JN242243 | (*E*)-nerolidol | Green et al., 2012 | Acc25053 | g | 7 | 22 | 573 | no | yes | yes |

**Table S2B.** Published and functionally characterised AcTPS genes and corresponding Red5 gene models.

Published name, GenBank accession number, main product and reference for published *Actinidia* TPS genes. Red5 Gene model: Best BLASTp hit to Red5 Gene models from the Genome Database (http://plants.ensembl.org/Actinidia_chinensis/Info/Index). Other column descriptions as per **Table S2A** above. AcTPS1a-d = *Actinidia chinensis* var. *chinensis* terpene synthase a-d; AdGDS = *Actinidia chinensis* var. *deliciosa* germacrene D synthase1; AdAFS1 = *Actinidia chinensis* var. *deliciosa* α-farnesene synthase1; AaLS1 = *A. arguta* linalool synthase1; AaTPS1 = *A. arguta* terpene synthase1; ApLS1 = *A. polygama* linalool synthase1; AcTPS1 = *A. chinensis* var. *chinensis* terpene synthase1; AcNES1 = *A. chinensis* var. *chinensis* nerolidol synthase1.

| **Abbreviation** | **Full gene name** | **GenBank accession** | |
| --- | --- | --- | --- |
| AmMs | *Antirrhinum majus* myrcene synthase | | AAO41727 |
| AmNES | *Antirrhinum majus* nerolidol synthase | | ABR24417 |
| AtCPS1 | *Arabidopsis thaliana* ent-copalyl diphosphate synthase | | NP_192187 |
| AtTPS03 | *Arabidopsis thaliana* terpene synthase 3 | | At4g16740 |
| AtTPS10 | *Arabidopsis thaliana* terpene synthase 10 | | AAC39443 |
| AtTPS14 | *Arabidopsis thaliana* terpene synthase 14 | | NP176361 |
| CmCPS  CsCPS | *Cucurbita maxima* ent-copalyl diphosphate synthase  *Camellia sinensis* ent-copalyl diphosphate synthase | | AAD04292  XP_028116786 |
| CrGES | *Catharanthus roseus* geraniol synthase | | AFD64744 |
| CsFS | *Cucumis sativus* E,E-alpha-farnesene synthase | | AY640154 |
| CsKS1 | *Camellia sinensis* ent-kaurene synthase | | QNN26118 |
| CsRLIS | *Camellia sinensis* (R)-linalool synthase | | QNI69163.1 |
| FaNES2 | *Fragaria x ananassa* nerolidol synthase 2 | | CAD57081 |
| GmAFS | *Glycine max* α-farnesene synthase | | XP_003551095.1 |
| MdAFS1 | *Malus x domestica* α-farnesene synthase 1 | | AAX19772 |
| MdCAR | *Malus x domestica* caryophyllene synthase | | JX848729 |
| MdGDS | *Malus x domestica* germacrene-D synthase | | JX848730 |
| MdLIS | *Malus x domestica* linalool synthase | | JX848734 |
| MdNES | *Malus x domestica* nerolidol synthase | | JX848731 |
| MdOCS | *Malus x domestica* ocimene synthase | | JX848733 |
| MdPIN | *Malus x domestica* pinene synthase | | JX848732 |
| MpFS | *Mentha x piperita* (E)-beta-farnesene | | AAB95209 |
| MsLS | *Mentha spicata* limonene synthase | | AAC37366 |
| OeGES1 | *Olea europaea* geraniol synthase 1 | | AFI47926 |
| PcAFS1 | *Pyrus communis* α-farnesene synthase 1 | | AAT70237 |
| PpTPS1 | *Prunus persica* terpene synthase 1 | | Prupe.4G030400 |
| PpTPS2 | *Prunus persica* terpene synthase 2 | | Prupe.4G029900 |
| PaTPS-far | *Picea abies* E,E-α-farnesene synthase | | AAS47697 |
| PtCPS | *Populus trichocarpa* ent-copalyl diphosphate synthase | | BAA84918 |
| PtTPS2 | *Populus trichocarpa* terpene synthase 2 | | AEI52902 |
| SlTPS3 | *Solanum* *lycopersicum* terpene synthase 3 | | JN408284 |
| SlTPS4 | *Solanum* lycopersicum terpene synthase 4 | | JN408285 |
| SlTPS5 | *Solanum* lycopersicum terpene synthase 5 | | JN408286 |
| SlTPS7 | *Solanum lycopersicum* terpene synthase 7 | | JN408287 |
| SlTPS8 | *Solanum lycopersicum* terpene synthase 8 | | JN408288 |
| SlTPS9 | *Solanum lycopersicum* terpene synthase 9 | | JN408289 |
| SlTPS12 | *Solanum lycopersicum* terpene synthase 12 | | JN412092 |
| SlTPS17 | *Solanum lycopersicum* terpene synthase 17 | | JN412089 |
| SlTPS24 | *Solanum lycopersicum* terpene synthase 24 | | JN412086 |
| SlTPS37 | *Solanum lycopersicum* terpene synthase 37 | | JN412077 |
| SlTPS38 | *Solanum lycopersicum* terpene synthase 38 | | JN412076 |
| SlTPS39 | *Solanum lycopersicum* terpene synthase 39 | | JN412075 |
| SlTPS40 | *Solanum lycopersicum* terpene synthase 40 | | JN412074 |
| SrCPS | *Stevia rebaudiana* ent-copalyl diphosphate synthase | | AAB87091 |
| VvTPS56 | *Vitis vinifera* terpene synthase 56 | | LOC100266449 |
| ZmTPS1 | *Zea mays* terpene synthase 1 | | AAO18435 |
| AaTPS1 | *Actinidia arguta* terpene synthase 1 | | KF319036 |
| AaLS1 | *Actinidia arguta* linalool synthase1 | | GQ338153 |
| AcAFS1 | *Actinidia chinensis* var. *chinensis* α-farnesene synthase 1 | | PSS10265.1 |
| AcBCS | *Actinidia chinensis* var. *chinensis* caryophyllene synthase | | OM884052 |
| AcCPS | *Actinidia chinensis* var. *chinensis* ent-copalyl diphosphate synthase | | PSS14615**.**1 |
| AcEKS | *Actinidia chinensis* var. *chinensis* ent-kaurene synthase | | PSR92592.1 |
| AcGDS | *Actinidia chinensis* var. *chinensis* germacrene-D synthase | | PSS06307.1* |
| AcGES | *Actinidia chinensis* var. *chinensis* geraniol synthase | | OM884053 |
| AcLIS/NES | *Actinidia chinensis* var. *chinensis* linalool/nerolidol synthase | | OM884054 |
| AcLS1 | *Actinidia chinensis* var. *chinensis* linalool synthase 1 | | PSS04734.1 |
| AcNES1 | *Actinidia chinensis* var. *chinensis* nerolidol synthase 1 | | JN242243 |
| AcNES2 | *Actinidia chinensis* var. *chinensis* nerolidol synthase 2 | | OM884050 |
| AcNES3 | *Actinidia chinensis* var. *chinensis* nerolidol synthase 3 | | OM884051 |
| AcTPS1a | *Actinidia chinensis* var. *chinensis* terpene synthase 1a | | MK809270 |
| AcTPS1b | *Actinidia chinensis* var. *chinensis* terpene synthase 1b | | MK809271 |
| AcTPS1c | Actinidia chinensis var. *chinensis* terpene synthase 1c | | MK809272 |
| AcTPS1d | *Actinidia chinensis* var. *chinensis* terpene synthase 1d | | MK809273 |
| AcTPS1 | *Actinidia chinensis* var. *chinensis* terpene synthase 1 | | KF319035 |
| AdAFS1 | *Actinidia chinensis* var. *deliciosa* α-farnesene synthase 1 | | FJ265785 |
| AdGDS | *Actinidia chinensis* var. *deliciosa* germacrene-D synthase | | AY789791 |
| ApLS1 | *Actinidia polygama* linalool synthase1 | | GQ338154 |

**Table S3**. Abbreviation, full gene name and GenBank accession number for TPS used in this study.

* the AcGDS amino acid sequence (see below) used for phylogenetic analysis was derived from the Red5 gene model Acc19469 by extending it with 55 amino acids at the N-terminus (underlined) based on alignment with AdGDS.

**AcGDS**:

MALSCTQGLPIPTMTTKTSIEPPHVTRRSANYHPTVWGDHFLAYSSDAMEEGDINMEQQQRLFQLKQKVRKMLEAAAGQSSQMLNLVDKIQRLGVSYHFETEIETALQHIYETCDHHFDDLHTAALSFRLLRQQGYPVSCDMFDKFKNSKGEFQESIISDVQGMLSLYEATFLRIRGEDILDEALAFTTIQLQSALPNLSTPIKEQIIHALNQPIHKGLTRLNARSHILFSEQNDCHSKDLLNFAQLDFNLLQKLHQRELYEITRWWKDLNFAKKLPFARDRISECYFWILGVYFEPQYLVARRMLTKVIAMISIIDDIYDVYGTLEELVLFTDAIERWEISALDQLPEYMKLCYQALLDVYSMIDEEMAKQGRSYCVDYAKSSMKSLVRAYFEEAKWFHQGYVPTMEEYMQVAIVTGAYKILATTSFVGMGELATKEVFDWVSNDPLIVQAASIVSRLTDDIVGHKFEQNRGHVASAVECYMKQHGTTEEEAIVELYKQVTNAWKDMNAECLFPTKVPMPLLVRVLNLARVINVLYKDEDGYTHSRTKVKKFVTSVLVDFVPIS

| **Terpenes** | **Id** | **Mean** | **SE** | **%** | **Mean** | **SE** | **%** | **Mean** | **SE** |
| --- | --- | --- | --- | --- | --- | --- | --- | --- | --- |
|  |  | **AcLIS/NES + DXS** | | | **AcLIS/NES + GUS** | | | **GUS + DXS** | |
| linalool | RTI, St | 3995.2 | 334.8 | 92.5 | 137.3 | 11.8 | 95.7 | 1.1 | 0.2 |
| linalool oxide (*cis*-furanoid) | RTI, St | 220.7 | 112.8 | 5.1 | 3.0 | 0.4 | 2.0 | 0.7 | 0.3 |
| linalool 3,7-oxide (*cis*) | RTI, St | 56.8 | 26.5 | 1.3 | 2.1 | 0.3 | 1.4 | / | / |
| linalool oxide (pyranoid)* | RTI | 16.4 | 8.2 | 0.3 | / | / | / | / | / |
| (*E*)-nerolidol | RTI, St | 9.2 | 4.7 | 0.2 | 0.5 | 0.1 | 0.3 | 0.2 | / |
|  |  | **AcLIS/NES + HMGR** | | | **AcLIS/NES + GUS** | | | **GUS + HMGR** | |
| linalool | RTI, St | 140.9 | 34.0 | 66.3 | 137.3 | 11.8 | 99.3 | 0.4 | 0.1 |
| α-muurolene | RTI | 35.9 | 6.5 | 16.9 | 0.5 | 0.02 | 0.3 | 40.4 | 4.8 |
| (*E*)-nerolidol | RTI, St | 35.6 | 8.7 | 16.7 | 0.5 | 0.1 | 0.3 | 0.1 | 0.02 |
|  |  | **AcNES2 + HMGR** | | | **AcNES2 + GUS** | | | **GUS + HMGR** | |
| (*E*)-nerolidol | RTI, St | 11844.0 | 1839.0 | 100 | 5223.0 | 3208.0 | 100 | 155.0 | 92.0 |
|  |  | **AcNES3 + HMGR** | | | **AcNES3 + GUS** | | | **GUS + HMGR** | |
| (*E*)-nerolidol | RTI, St | 586.0 | 228.2 | 100 | 28.2 | 15.3 | 100 | 0.1 | 0.02 |
|  |  | **AcBCS + HMGR** | | | **AcBCS + GUS** | | | **GUS + HMGR** | |
| β-caryophyllene | RTI, St | 6100.0 | 596.0 | 82.3 | 386.0 | 48.0 | 80.9 | / | / |
| α-humulene | RTI, St | 403.0 | 46.0 | 5.4 | 24.0 | 3.8 | 4.9 | / | / |
| epi-cubebol | RTI | 120.0 | 10.4 | 1.6 | 12.4 | 2.5 | 2.6 | / | / |
| caryophyllene oxide (*cis*) | RTI | 141.0 | 15.0 | 1.9 | / | / | / | / | / |
| caryophyllene oxide (*trans*) | RTI | 610.0 | 88.0 | 8.2 | 55.0 | 2.3 | 11.6 | / | / |
|  |  | **AcGES + DXS** | | | **AcGES + GUS** | | | **GUS + DXS** | |
| geraniol | RTI, St | 12.9 | 3.7 | 89.6 | 1.3 | 0.5 | 95.6 | 2.3 | 0.2 |
| geranyl acetone | RTI, St | 1.5 | 0.2 | 10.4 | 0.6 | 0.1 | 4.4 | 0.8 | 0.2 |

*****2H-Pyran-3-ol, 6-ethenyltetrahydro-2,2,6-trimethyl-

**Table S4.** Complete dataset of headspace volatile terpenes produced by transient expression of AcTPS genes *in planta.*

*Nicotiana benthamiana* leaves were infiltrated with *Agrobacterium* suspensions containing the pHEX2 constructs indicated in combination with DXS (MEP pathway, monoterpene substrate) or HMGR (mevalonate pathway, sesquiterpene substrate). Headspace volatiles were collected by SPME 7 d post-infiltration and analysed by GC-MS. Mean and standard error (SE) are calculated on a ng∙gFW^-1^ basis using three biological replicates (n=3). Volatile terpenes found at 5-fold greater concentrations than the negative controls (GUS + DXS; GUS + HMGR) and representing greater than 0.2% of total terpene content are shown. Identification (Id) was by mass spectrum comparison and using the retention times and indices (RTI) from NIST, Wiley and in-house mass spectral libraries. St = identification confirmed by comparison with an authentic standards (St). ‘/’ = not detected. AcNES3 = AcTPS1; AcNES2 = AcTPS3, AcLIS/NES = AcTPS9/10; AcBCS = AcTPS5 and AcGES = AcTPS15.

|  |  | | |  | |  | **AcLIS/NES** | | **AcNES3** | **AcGES** | **AcNES2** | **AcBCS** |
| --- | --- | --- | --- | --- | --- | --- | --- | --- | --- | --- | --- | --- |
| **No.** | **Terpenes** | | | **RI** | | **ID** | **GDP** | **FDP** | **FDP** | **GDP** | **FDP** | **FDP** |
| **Monoterpenes** | | |  |  | |  |  |  |  |  |  |  |
| 1 | α-pinene | | | 1021 | | RTI, St | 0.09 | / | / | 0.01 | / | / |
| 2 | β-pinene | | | 1099 | | RTI, St | 0.12 | / | / | 0.02 | / | / |
| 3 | β-myrcene | | | 1157 | | RTI, St | 7.18 | / | / | 17.41 | / | / |
| 4 | limonene | | | 1194 | | RTI, St | 0.72 | / | / | / | / | / |
| 5 | β-phellandrene | | | 1202 | | RTI, St | 0.37 | / | / | 2.45 | / | / |
| 6 | β-ocimene | | | 1233 | | RTI | 3.59 | / | / | / | / | / |
| 7 | γ-terpinene | | | 1245 | | RTI, St | 0.21 | / | / | / | / | / |
| 8 | terpinolene | | | 1284 | | RTI, St | 0.28 | / | / | / | / | / |
| 9 | *cis*-linaloloxide | | | 1471 | | RTI, St | 0.01 | / | / | / | / | / |
| 10 | linalool | | | 1549 | | RTI, St | 87.39 | / | / | 80.55 | / | / |
| 11 | geraniol | | | 1853 | | RTI, St | / | / | / | / | / | / |
| **Sesquiterpenes** | |  | | |  |  |  |  |  |  |  |  |
| 12 | δ-cadinene | | | 1063 | | RTI | / | / | / | / | / | / |
| 13 | cubenene | | | 1080 | | RTI | / | / | / | / | / | / |
| 14 | β-caryophyllene | | | 1580 | | RTI, St | / | / | / | / | / | 100 |
| 15 | (*E*)*-*β-famesene | | | 1664 | | RTI, St | / | 27.36 | 8.74 | / | 2.25 | / |
| 16 | (*Z,Z*)*-*α-farnesene | | | 1692 | | RTI | / | / | 0.12 | / | 0.26 | / |
| 17 | (*Z,E*)*-*α-farnesene | | | 1724 | | RTI | / | / | 3.03 | / | 0.22 | / |
| 18 | α-farnesene | | | 1749 | | RTI, St | / | 8.33 | 5.58 | / | 2.81 | / |
| 19 | (*E*)-nerolidol | | | 2041 | | RTI, St | / | 64.32 | 83.2 | / | 94.46 | / |

**Table S5.** Volatile terpenes produced by heterologous expression of AcTPS genes in *E. coli.*

Recombinant enzymes were purified by Ni^2+^ affinity and gel filtration chromatography, and were incubated with GDP or FDP as substrate. Terpene volatiles were collected by SPME and analyzed by GC-MS in triplicate. Each terpene is expressed as a percentage of total terpenes identified. ‘/’ = not detected. Identification (Id) was by comparison with mass spectra, retention times and indices (RTI) from NIST, Wiley and in-house mass spectral libraries. St = identification confirmed by comparison with an authentic standards (St).

**S6A** Terpenes produced by *Actinidia* leaves after methyl jasmonate (JA) treatment (terpene accumulation)

| **Terpenes (ng∙g^-1^)** | **Hort16A**  **+ JA** | **Hort16A**  **+ Buffer** | **Hayward**  **+ JA** | **Hayward**  **+ Buffer** |
| --- | --- | --- | --- | --- |
| β-ocimene | 56.7±12.9 | 26.2±4.8 | 105.4±22.5 | 129.8±24.6 |
| linalool | 1310.9±90.6 | 936.5±41.0 | 1845.1±222.7 | 2061.1±265.7 |
| α-terpineol | 87.1±27.5 | 161.1±84.9 | 32.4±12.4 | 59.8±9.9 |
| citronellol | 45.0±2.5 | 45.1±8.6 | 116.67±9.4 | 116.1±15.4 |
| geraniol | 103.6±11.0 | 40.8±15.8 | 140.9±60.0 | 188.2±32.2 |
| β-myrcene | 153.1±54.2 | 138.7±29.8 | 32.6±37.3 | 10.2±9.4 |
| D-limonene | 17.2±8.7 | 30.1±8.0 | / | / |
| *trans*-linalool oxide | 110.2±52.4 | 75.8±14.8 | 7.2±12.4 | / |
| terpinolene | 7.6±6.9 | 4.2±7.2 | 2.6±4.4 | 1.4±2.4 |

**S6B** Terpenes produced by *Actinidia* leaves after methyl jasmonate (JA) treatment (terpene emission)

| **Terpenes (ng∙g^-1^)** | **Hort16A**  **+ JA** | **Hort16A**  **+ Buffer** | **Hayward**  **+ JA** | **Hayward**  **+ Buffer** |
| --- | --- | --- | --- | --- |
| β-ocimene | 14.5±1.7 | / | 21.1±2.9 | / |
| DMNT | 1107.9±513.2 | / | 561.5±241.5 | / |
| linalool | 83.6±15.5 | / | / | / |
| β-caryophyllene | 174.2±60.8 | / | / | / |
| β-pinene | 16.4±1.6 | 15.1±2.6 | 11.2±1.2 | 10.3±0.5 |

**S6C** Terpenes produced by *Actinidia* leaves after Brown-headed Leaf Roller infestation (terpene accumulation)

| **Terpenes (ng.g^-1^)** | **Hort16A**  **+ BHLR** | **Hort16A**  **- BHLR** | **Hayward**  **+ BHLR** | **Hayward**  **- BHLR** |
| --- | --- | --- | --- | --- |
| linalool | 2834.4±91.4 | 2145.8±286.7 | 2741.3±141.6 | 1650.1±178.8 |
| α-terpineol | 13.9±3.2 | 30.7±9.1 | 16.3±1.1 | 21.8±1.2 |
| geraniol | 28.5±3.1 | 27.9±4.4 | 25.9±2.6 | 17.9±1.8 |
| cis-linaloloxide | 37.1±4.2 | 3.1±0.2 | 27.8±6.9 | 9.9±0.6 |
| (*E*)-nerolidol | 5.8±4.1 | 5.6±0.2 | 0 | 0 |

**S6D** Terpenes produced by *Actinidia* leaves after Brown-headed Leaf Roller infestation (terpene emission)

| **Terpenes (ng∙g^-1^)** | **Hort16A**  **+ BHLR** | **Hort16A**  **- BHLR** | **Hayward**  **+ BHLR** | **Hayward**  **- BHLR** | **BHLR** |
| --- | --- | --- | --- | --- | --- |
| linalool | 667.0±123.2 | 0.8±0.4 | 502.3±58.7 | 0.7±0.4 | 0.2±0.1 |
| (*E*)-nerolidol | 537.7±271.6 | 0.3±0.2 | 691.5±626.7 | 0.4±0.1 | 0.2±0.1 |
| (*Z*)-nerolidol | 397.0±184.4 | 0.6±0.4 | 512.5±435.0 | 0.7±0.5 | 0.3±0.2 |

**Table S6:** Terpenes (accumulation and emission) produced by *Actinidia* leaves after methyl jasmonate treatment (**S6A, B**) or herbivory by Brown-headed Leaf Roller (**S6C, D**).

| Primer name | Sequence (5’-3’) | | | |
| --- | --- | --- | --- | --- |
| ***Amplification from cDNA and gateway cloning into pHEX2 for transient expression in planta*** | | | | |
| AcNES2-F | GGGGACAAGTTTGTACAAAAAAGCAGGCTATGTCAAAAACCAATGAGTC | | | |
| AcNES2-R | GGGGACCACTTTGTACAAGAAAGCTGGGTTTACTCTTCTTCATGACTGC | | | |
| AcNES3-F | GGGGACAAGTTTGTACAAAAAAGCAGGCTATGGAGTCCTCACTAGCA | | | |
| AcNES3-R | GGGGACCACTTTGTACAAGAAAGCTGGGTTTAGGAGGTAGGGACTAG | | | |
| AcGES-F | GGGGACAAGTTTGTACAAAAAAGCAGGCTATGGCTTGCATGAAGAGCC | | | |
| AcGES-R | GGGGACCACTTTGTACAAGAAAGCTGGGTTTAAAAGCGAATGGATGTGAAGA | | | |
| AcBCS-F | GGGGACAAGTTTGTACAAAAAAGCAGGCTATGGCAATATCATGTGCTAC | | | |
| AcBCS-R | GGGGACCACTTTGTACAAGAAAGCTGGGTCTAATTGATTGGTACAGAATC | | | |
| AcLIS/NES-F | GGGGACAAGTTTGTACAAAAAAGCAGGCTATGGAATTTGTTCTTTCATATAACTCT | | | |
| AcLIS/NES-R | GGGGACCACTTTGTACAAGAAAGCTGGGTTCATTTGATTGGAATTGGATCCA | | | |
| ***For cloning in pET300/NT-DEST and creating His-tagged recombinant protein*** | | | | |
| AcNES2-F | GGGGACAAGTTTGTACAAAAAAGCAGGCTCCATGTCAAAAACCAATGAGTC | | | |
| AcNES2-R | GGGGACCACTTTGTACAAGAAAGCTGGGTTTACTCTTCTTCATGACTGC | | | |
| AcNES3-F | GGGGACAAGTTTGTACAAAAAAGCAGGCTCCATGGAGTCCTCACTAGCA | | | |
| AcNES3-R | GGGGACCACTTTGTACAAGAAAGCTGGGTTTAGGAGGTAGGGACTAG | | | |
| AcGES-F | GGGGACAAGTTTGTACAAAAAAGCAGGCTCCATGGCTTGCATGAAGAGCC | | | |
| AcGES-R | GGGGACCACTTTGTACAAGAAAGCTGGGTTTAAAAGCGAATGGATGTGAAGA | | | |
| AcBCS-F | GGGGACAAGTTTGTACAAAAAAGCAGGCTCCATGGCAATATCATGTGCTAC | | | |
| AcBCS-R | GGGGACCACTTTGTACAAGAAAGCTGGGTCTAATTGATTGGTACAGAATC | | | |
| ***For cloning into pMalc6t and creating Mal-tagged recombinant protein*** | | | | |
| AcLIS/NES-NotI-F | TTAAGCGGCCGCATGGAATTTGTTCTTTCATATAACTCT | | | |
| AcLIS/NES-EcoRI-R | TTAAGAATTCTCATTTGATTGGAATTGGATCCA | | | |
| Primer name | | Sequence (5’-3’) | Product size (bp) | Primer efficiency |
|  | | ***For qRT-PCR*** |  |  |
| Acc09580-QF (AcNES3) | | CATGGCTGACGGAATCAACG | 86 | 2 |
| Acc09580-QR | | AGCAATAGAGGTCATGCCCG |  |  |
| Acc13525-QF (AcNES2) | | GTGGATGGACTTGGGCTTGA | 109 | 1.958 |
| Acc13525-QR | | GTAATTGGAGAAGCGGGGCT |  |  |
| Acc13742-QF (AcBCS) | | GATCGCTAGGCTCACGGATG | 103 | 1.958 |
| Acc13742-QR | | TCATGTAGCATTCAACTGCCGA |  |  |
| Acc17359-QF (AcAFS1) | | AGCTGCATCAATTGTTTCCAGAC | 95 | 2 |
| Acc17359-QR | | CATGTAGCATTCAACCGCCG |  |  |
| Acc19057-58-QF (AcLIS/NES) | | CATATTTGGTGGAAGCGCGG | 89 | 1.994 |
| Acc19057-58-QR | | ACCGCCTACTGAAACCCATG |  |  |
| Acc19649-QF (AcGDS) | | AGTCCGCATTGCCCAACTTA | 101 | 2 |
| Acc19649-QR | | GCTCCTTGCATTGAGCCTTG |  |  |
| Acc20592-QF (AcLS1) | | AGCCTAGCGTCATTCATGGAA | 99 | 2 |
| Acc20592-QR | | AGTTAAGTCCATTTTTGCAAGTTCTTG |  |  |
| Acc25053-QF (AcNES1) | | GCTATATGAAAGAGCACAAGGGC | 100 | 1.93 |
| Acc25053-QR | | AGGCATTCCTTGTTGAGGCA |  |  |
| Acc26061-QF (AcGES) | | AGCAAGAACGAGGAGACGTG | 108 | 2 |
| Acc26061-QR | | AGCCACCGATGAGTCGTTTT |  |  |
| AcTPS1a-QF | | CAAGGCGTACCCTTCGATGT | 89 | 2 |
| AcTPS1a-QR | | GGAAGGTTGGTAGGATGCC |  |  |
| AcTPS1b-QF | | CAAGGCGTACCCTTCGATGT | 89 | 2 |
| AcTPS1b-QR | | AGAAGGTTGGTAGGATGCG |  |  |
| AcTPS1c-QF | | GTCTGCGTATCCTTAGATGCAG | 126 | 2 |
| AcTPS1c-QR | | CACTATTTAGAGATTGAAC |  |  |
| AcTPS1d-QF | | GCACAAGCAAAACTGCCC | 81 | 2 |
| AcTPS1d-QR | | CGGAACCTAGCAAAATTAAC |  |  |
| Adactin-QF | | CCAAGGCCAACAGAGAGAAG | 197 | 1.958 |
| Adactin-QR | | GACGGAGGATAGCATGAGGA |  |  |
| Ef1a-QF | | GCACTGTCATTGATGCTCCT | 118 | 1.968 |
| Ef1a-QR | | CCAGCTTCAAAACCACCAGT |  |  |

**Table S7.** Primers used in this study.

Restriction sites and attB1 and attB2 sequences in primers used for cloning are underlined. Gateway cloning reactions were performed as recommended by the manufacturer (Invitrogen). Vector references: pHEX2 (Hellens *et al*., 2005), pET300/NT-DEST (https://www.thermofisher.com) and pMalc6t (Invitrogen).

**References:**

**Christianson DW.** 2006. Structural biology and chemistry of the terpenoid cyclases. *Chemical Reviews* **106**, 3412-3442 doi: 10.1021/cr050286w.

**Zhou K, Peters RJ.** 2009. Investigating the conservation pattern of a putative second terpene synthase divalent metal binding motif in plants. *Phytochemistry* **70**, 366-369 doi: 10.1016/j.phytochem.2008.12.022.
