# Supplementary material for: The terpene synthase (TPS) gene family in kiwifruit shows high functional redundancy and a subset of TPS likely fulfil overlapping functions in fruit flavour, floral bouquet and defence
Source: Mol Hortic. 2023 May 8;3:9. doi: 10.1186/s43897-023-00057-0 (PMC10514967; doi:10.1186/s43897-023-00057-0)
Supplement: Supplementary file 2 — Additional file 2: Figure S1. Cloning strategy for AcGES and AcLIS/NES based on RNAseq coverage and gene models. Figure S2. Amino acid alignment of full length AcTPS genes identified in the Red5 genome. Figure S3. SDS-PAGE analysis of purified recombinant His-tagged and Mal-tagged AcTPS proteins. Figure S4. Experimental setup for herbivore treatment of kiwifruit leaves. Figure S5. Hormone treatment of young Red5 fruit. [file 43897_2023_57_MOESM2_ESM.docx]

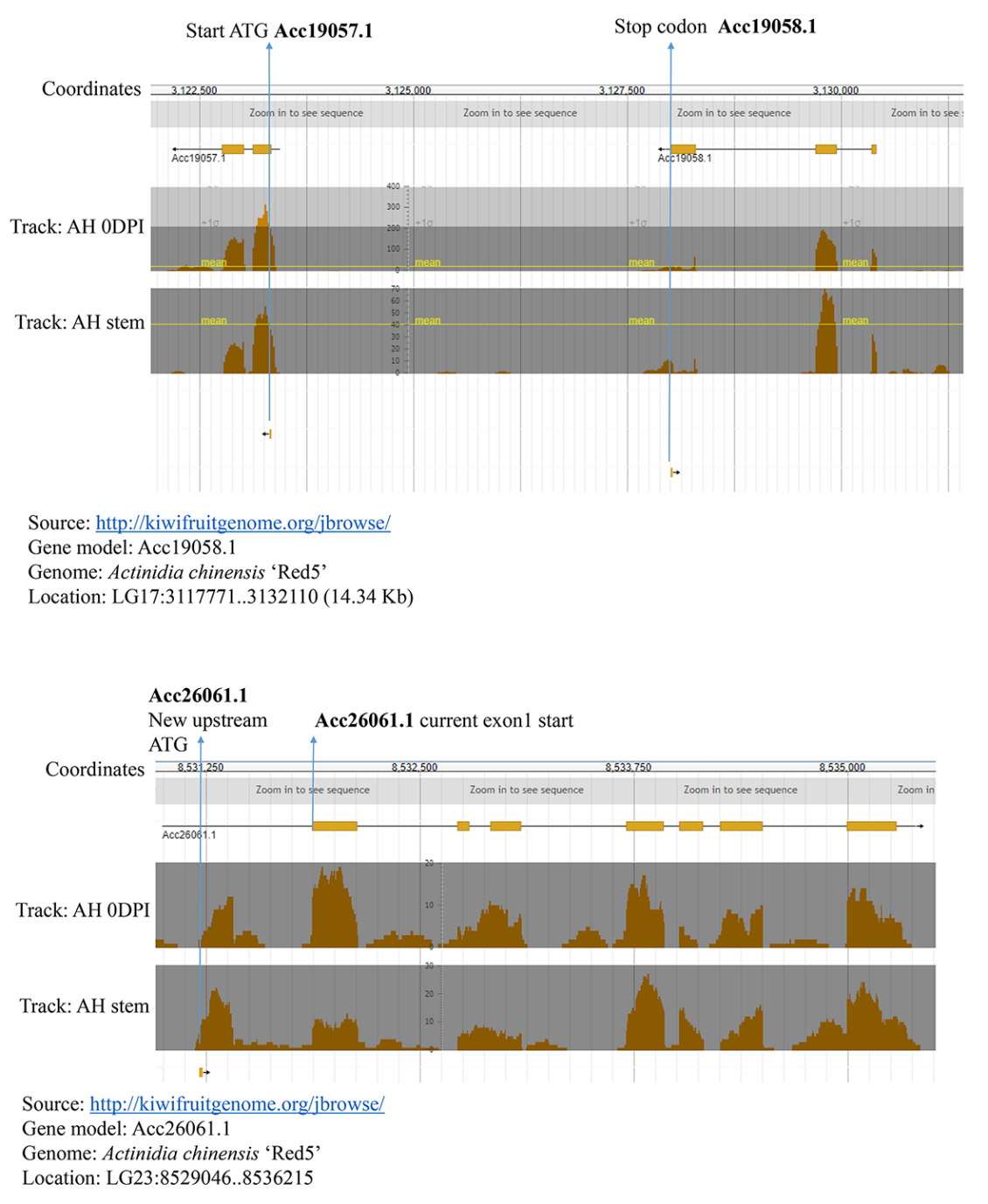


**Figure S1**. Cloning strategy for *AcLIS/NES* and *AcGES* based on RNAseq coverage (tracks) and gene models (yellow boxes).

**Upper panel**: Acc19057.1 and Acc19058.1 were combined to design forward (Start ATG) and reverse (Stop codon) primers and resulted in the cloning of *AcLIS/NES*. **Lower panel**: A new ATG start codon located upstream relative to the Acc26061.1 “current exon1” gene model prediction was identified for *AcGES* (labelled as “New upstream ATG”) by studying RNAseq data alignment tracks.

**
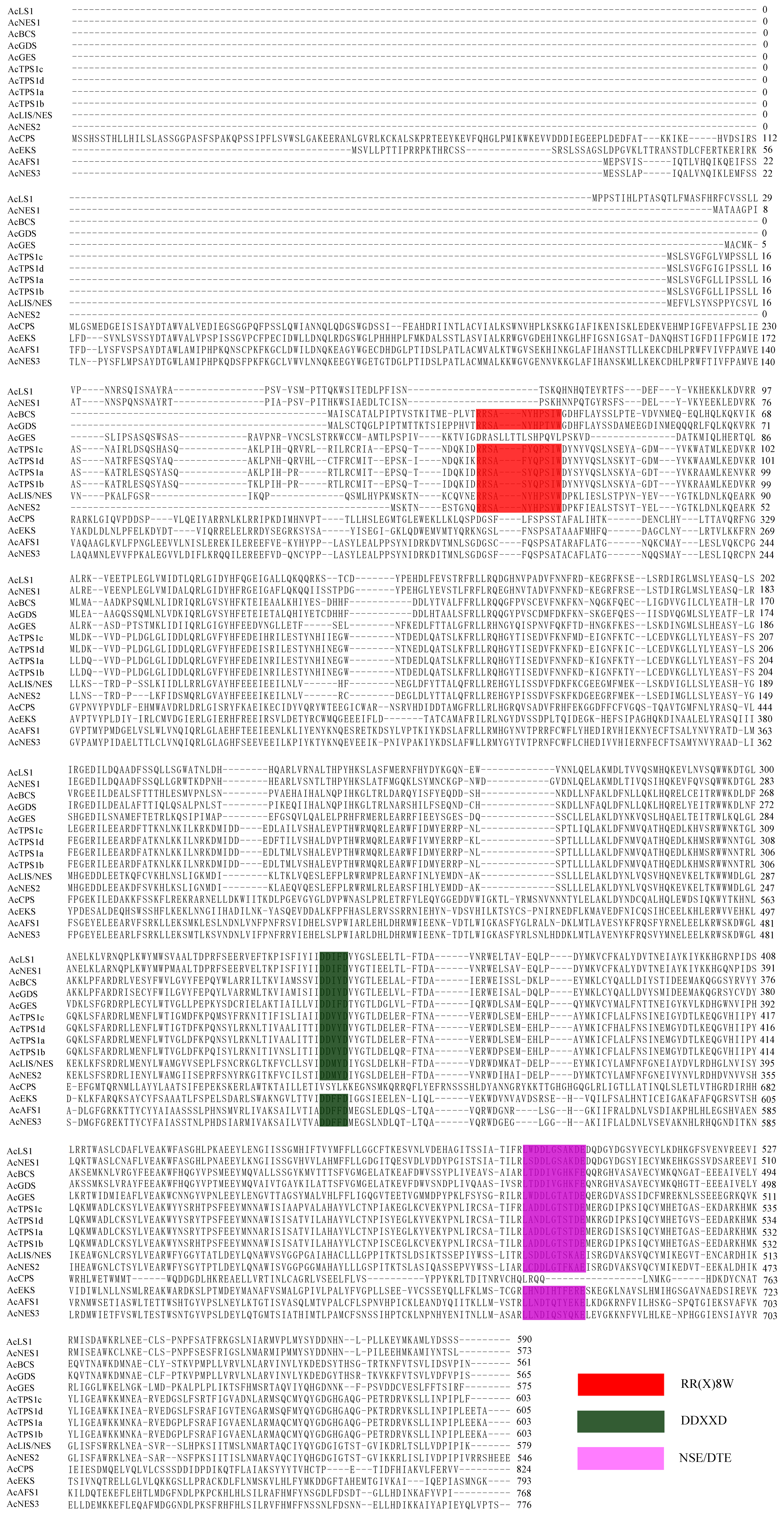
**

**Figure S2**. Amino acid alignment of full-length AcTPS genes identified in the Red5 genome. The conserved motifs RR(X)8W, DDXXD and NSE/DTE are highlighted with red, green and pink background respectively. The NSE/DTE metal binding motif is based on the consensus: (L,V)(V,L,A)(N,D)D(L,I,V)x(S,T,G)xxxE (Christianson, 2006; Zhou and Peters, 2009).


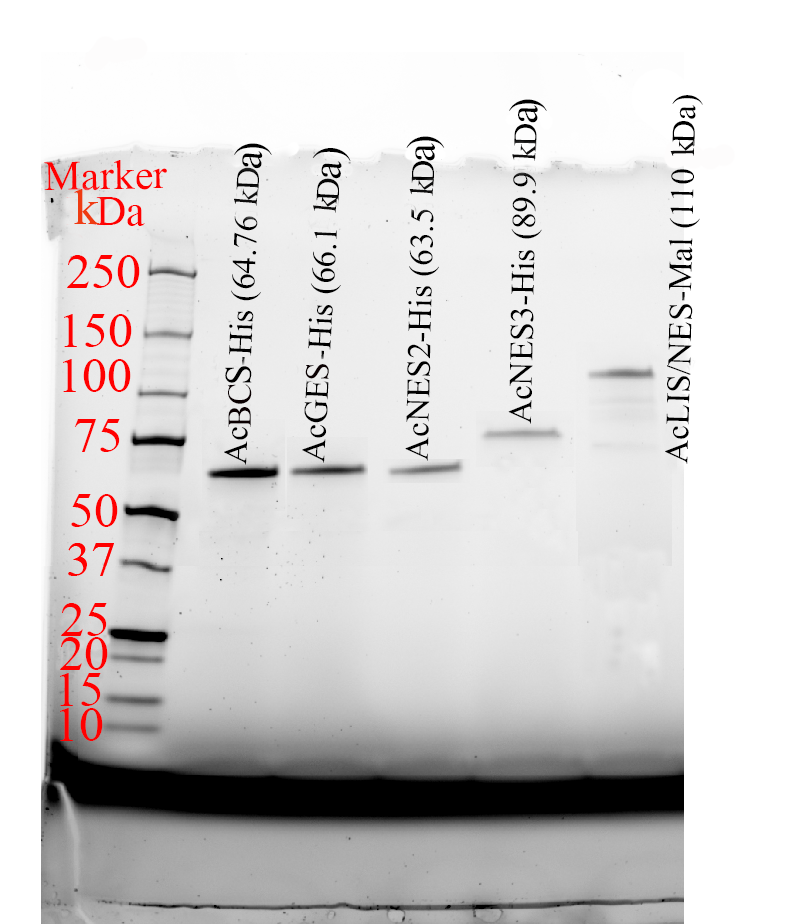


**Figure S3**. SDS-PAGE analysis of purified recombinant His-tagged and Mal-tagged AcTPS proteins. The Precision Plus Protein^TM^ Unstained marker (10 µL/lane) was purchased from Bio-Rad Laboratories (Bio-Rad, USA).


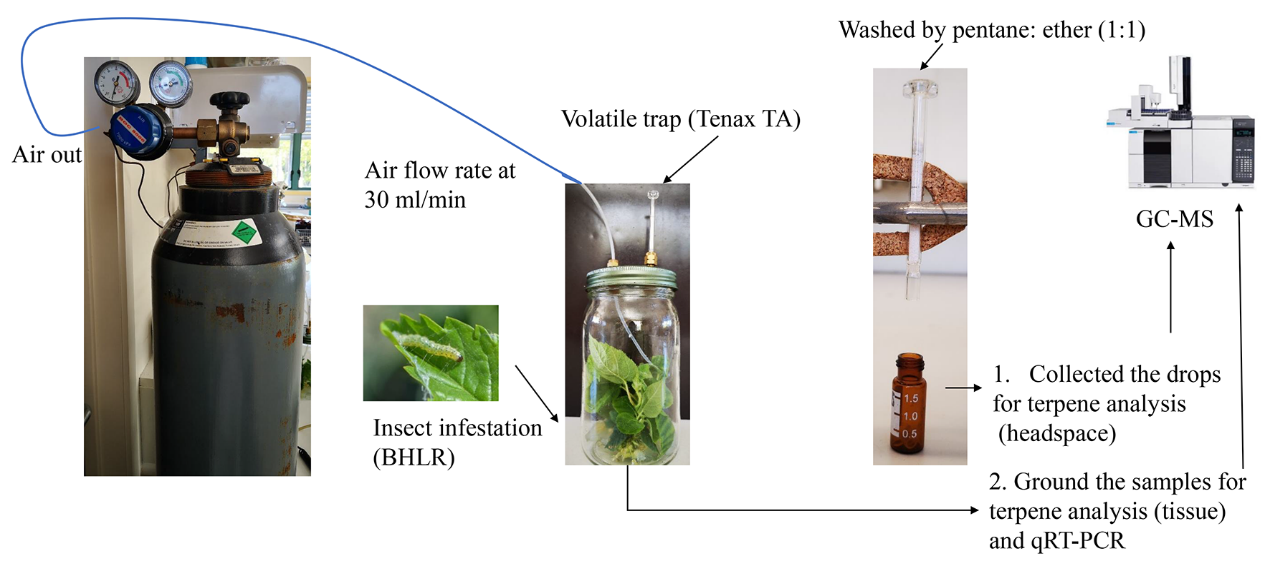


**Figure S4**. Experimental set-up for herbivore treatment of kiwifruit leaves.

‘Hort16A’ and ‘Hayward’ tissue culture grown plants were infested with brown-headed leafroller caterpillars of instar 3 inside a 1 L sealed jar and the released volatiles were trapped with a Tenax TA cartridge for 4–6 d. After elution of the trapped volatiles with pentane:ether (1:1), samples were analysed by GC-MS. In parallel, at the completion of the experiment, leaf samples were harvested and snap frozen for solvent extraction of terpenes present in the tissue by GC-MS analysis and for RNA/qRT-PCR gene expression analysis.


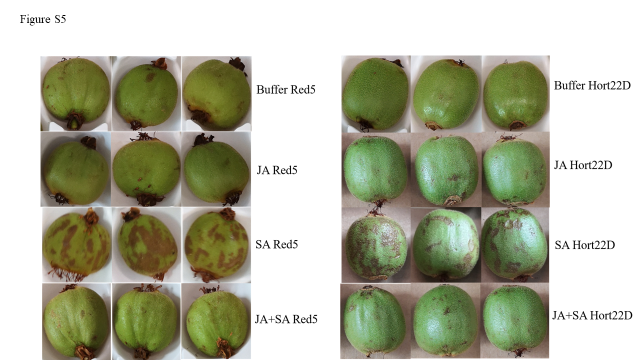


Buffer

JA

SA

JA + SA

**Figure S5.** Hormone treatment of young Red5 fruit.

*A. chinensis* var. *chinensis* ‘Red5’ were harvested at 45 d post anthesis and treated with methyl jasmonate (JA), salicylic acid (SA) or a combination of both (JA + SA). Photographs were taken 24 h post treatment compared to a buffer-treated control.

**References**

**Christianson DW.** 2006. Structural biology and chemistry of the terpenoid cyclases. *Chemical Reviews* **106**, 3412-3442 doi: 10.1021/cr050286w.

**Zhou K, Peters RJ.** 2009. Investigating the conservation pattern of a putative second terpene synthase divalent metal binding motif in plants. *Phytochemistry* **70**, 366-369 doi: 10.1016/j.phytochem.2008.12.022.
